# Copper-Catalyzed Dimerization/Cyclization of Itaconates

**DOI:** 10.3390/molecules200815023

**Published:** 2015-08-17

**Authors:** Zhiqiang Li, Ruirui Li, Lan Jiang, Zhengning Li

**Affiliations:** College of Environmental and Chemical Engineering, Dalian University, Dalian 116622, China; E-Mails: zqli501@163.com (Z.L.); lrrnice@126.com (R.L.); jianglan@dlu.edu.cn (L.J.)

**Keywords:** copper, catalysis, domino reaction, itaconate, dimerization, cyclopentanone

## Abstract

A copper-catalyzed domino reaction between itaconate esters and diethyl zinc (or silane) is developed, affording itaconate dimerization products, multi-ester-substituted cyclopentanones, in moderate to high yields.

## 1. Introduction

The metal-catalyzed conjugate addition of organic zinc reagents, which are mild alkylating reagents, to α,β-unsaturated carbonyls and esters, has been used extensively for C–C bond formation in organic synthesis [1,2,3,4,5,6,7]. The enolate intermediate formed in this process or by reductive addition can further react with an electrophile under the same reaction conditions, leading to a domino mode of reaction, which is highly desirable in organic synthesis. Applications of the conjugate reduction-derivated enolates have been reported in Negishi coupling carboannulation [8], aldolization [9,10], the Mannich reaction [11,12,13], and Ireland rearrangement [14].

**Scheme 1:**
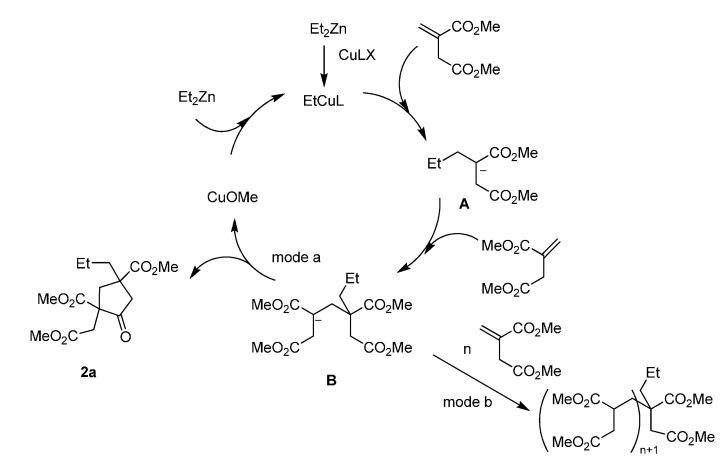
Reaction pathways for formation of **2a**.

**Scheme 2:**
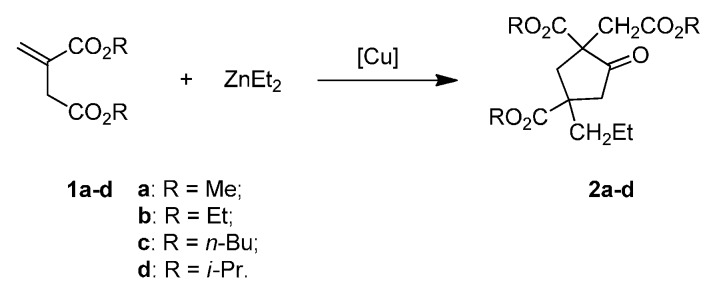
Domino reactions between itaconate esters and diethyl zinc.

Recently, we reported a copper-promoted conjugate reductive-aldol/lactonization domino reaction of dimethyl itaconate with a silane and carbonyls [15], and reductive Mannich reaction/lactamization of dimethyl itaconate with imines [16]. As a replacement of a silane reductant, we employed diethyl zinc as a nucleophile. In this course, a reaction of diethyl zinc with dimethyl itaconate (**1a**) was performed, yielding 2,4-bis(methoxycarbonyl)-2-(2-methoxy-2-oxoethyl)-4-propylcyclopentanone (**2a**), a multi-ester-substituted cyclopentanone. Of special interest is the richness of functional groups in **2a**. Both the ketone and ester functional groups in **2a** [17] and in other cyclopentanones bearing CO_2_R/CH_2_CO_2_R groups [18,19,20,21] can be converted to other functional groups, making **2a** and analogues valuable in organic synthesis. A literature survey indicated that there are only a few reports concerning the synthesis of this kind of molecules. A 21% and 26% yield of **2a** and 2,4-bis(methoxycarbonyl)-2-(2-methoxy-2-oxoethyl)-4-pentylcyclopentanone were obtained via a copper-catalyzed reaction of **1a** with ethylaluminum dichloride [17] and *n*-butyl magnesium bromide [22], respectively. Using [Rh(COD)Cl]_2_ as a catalyst, a 10% yield of substituted cyclopentanone was produced, along with 60% yield of the conjugate product benzyl succinate, via reaction of itaconate with phenylzinc chloride [1]. Given to the existence of two tertiary carbons in the product **2a**, the low yields reported in literature, and low price of copper catalysts, an efficient and concise synthesis of **2a** and alike compounds using copper catalysts offers very attractive prospectives. Herein, we wish to report that high yields could be achieved via double conjugate addition/cyclization domino reaction (Scheme 1) of itaconates with diethyl zinc (Scheme 2).

## 2. Results and Discussion

Reactions of diethyl zinc with **1a** were performed under various conditions, under the assumption that copper catalyzed the conjugate addition of ethyl anion to itaconate, an α,β-unsaturated ester, and the enolate **A** thus generated reacted with another itaconate to give enolate **B**, which cyclized to afford the cyclopentanone framework and copper methoxide (Scheme 1). The results are summarized in Table 1. Using diethyl zinc as the alkylating reagent, the proposed double conjugate addition/cyclization domino reaction proceeded even in the absence of an additional catalyst, affording the cyclopentanone **2a** in 40% yield with preference for the *trans*-isomer (Table 1, entry 1) [23,24]. Thus indicates the superiority of diethyl zinc over the aluminum reagent used in the literature [17]. Using Cu(OAc)_2_·2H_2_O, CuCl or CuBr as the catalyst, the yield reached 55%–65% with almost no diastereoselectivity (entries 2–4). An increase of the yield and no change of the diastereomeric ratio were observed using CuI catalyst at 25 °C (entry 5). *Trans*-**2a**, assigned by its NOSEY spectrum in Supplementary Materials), was formed as the dominant diastereomer at lower temperature (73% at −30 °C, entry 7).

**Table 1:** Reaction of diethyl zinc with dimethyl itaconate ^a^.

Entry	Cat.	Ligand	Temp (°C)	Time (h)	Yield ^b^ (%)	*cis*/*trans* ^c^
1	-	-	25	2.5	40	38:62
2	Cu(OAc)_2_·2H_2_O	-	25	4.5	56	52:48
3	CuCl	-	25	2.0	55	46:54
4	CuBr	-	25	2.0	65	55:45
5	CuI	-	25	2.0	75	37:63
6	CuI	-	−15	2.0	61	30:70
7	CuI	-	−30	2.0	68	27:73
8	CuFL	-	25	2.0	72	61:39
9	CuFL	-	0	2.5	73	57:43
10	CuFL	-	−30	9.0	67	62:38
11	CuFL	-	−58	21.0	40	56:44
12	CuFL	-	−78	21.0	1	-
13	CuFL ^d^	-	25	4.0	53	55:45
14	CuFL ^e^	-	25	2.5	74	62:38
15	CuFL	DPPP ^f^	25	2.0	38	53:47
16	CuFL	DPBen ^f^	25	2.0	89	62:38
17	CuFL	DPEphos ^f^	25	2.0	88	63:37
18	CuFL	Xantphos ^f^	25	2.0	95(85 ^g^)	62:38
19	CuFL ^h^	Xantphos	25	2.5	98	64:36

^a^ CuFL = CuF(PPh_3_)_3_·2MeOH, **1a** 1.6 mmol, **1a**:Et_2_Zn:[Cu] = 1.0:0.75:1.0% (molar ratio), toluene as the solvent unless noted; ^b^ GC yield; ^c^ determined by GC analysis; ^d^ THF as the solvent; ^e^ dichloromethane as the solvent; ^f^ 1.0 mol % ligand; ^g^ isolated yield; ^h^ **1a** 10 mmol, **1a**:Et_2_Zn:CuXL:Xantphos = 1.0:0.9:1.0%:1.0% (molar ratio).

CuF(PPh_3_)_3_·2MeOH, with good solubility and high activity in catalyzing conjugate addition and the subsequent reaction [25,26,27], was employed to catalyze the reaction of **1a**, and a 72% yield of **2a** was obtained. Interestingly, the *cis*-**2a** isomer was the dominant one with a 61:39 *cis*-/*trans*-ratio (entry 8). Efforts to improve the diastereoselectivity using this catalyst at lower reaction temperatures (entries 9–12) were not very successful. In the meanwhile, the yields decreased. Since itaconate was consumed and almost no by-products were observed by GC, it is reasonable to assume that the by-polymerization reaction was favored by decreasing the reaction temperature. This could be attributed to the competitive reactions of enolate **A**, either cyclizing to yield **2** (mode a in Scheme 1) or undergoing conjugate addition to itaconate to yield polymer (mode b in Scheme 1). Anion-induced polymerization of α,β-unsaturated esters, which involves conjugate addition of enolates to α,β-unsaturated esters, has been reported [28]. The phenomenon that cyclization was more sensitive to temperature than conjugate addition was also observed by Shibasaki in the formation of lactams [11]. Switching the solvent to THF and dichloromethane did not show any advantages, either in terms of yield or the diastereomeric ratio (entries 13 and 14).

It should be noted that the yield is affected by the mode of addition of the reactants. Addition of diethyl zinc to a mixture of **1a** and CuF(PPh_3_)_3_·2MeOH catalyst gave much higher yield of **2a** addition of **1a** to a mixture of diethyl zinc and the catalyst, which indicates that there is not much difference between the rate constants of intermediate **A** and **B**, and that formation of **B** is favorable at a high concentration ratio of **1a** to diethyl zinc. Using *n*-butylzinc chloride as an alkylating reagent, the monoconjugate addition product was obtained as a dominant one. This is similar to Frost’s result using phenylzinc chloride as a nucleophile under rhodium catalysis [1]. Dimethyl zinc, a less reactive reagent [29], was also tested to initialize the domino reaction, but the reaction did not proceed.

CuF(PPh_3_)_3_·2MeOH-diphosphorous catalysts, which have been mentioned in the literature as being advantageous in catalyzing conjugate reductions [30,31], were applied to this reaction. The introduction of 1,3-bis(diphenylphosphino)propane (dppp) decreased the yield (entry 15), while introducing 1,2-bis(diphenylphosphino)benzene (DPBen), 2,2′-bis(diphenylphosphinophenyl)ether (DPEphos), and 4,5-bis(diphenylphosphino)-9,9-dimethylxanthene (Xantphos) could increase the yield remarkably. Nevertheless, there was no great improvement of diastereomeric ratio (entries 16–18). Among the ligands screened, Xantphos gave a 95% yield using 1.6 mmol of dimethyl itaconate and a 98% yield at 10 mmol of dimethyl itaconate scale (entries 18 and 19).

Previous results indicated that using bulky alkyl α,β-unsaturated esters gave higher diastereoselectivity in reductive aldol domino reactions [26]. Accordingly, we resorted to bulky alkyl groups in the itaconate. Gratifyingly, improvement of the *cis-*/*trans-*ratios was achieved using diethyl itaconate, di-*n*-butyl itaconate and di-*i*-propyl itaconate (Table 2, entries 2–4). However, the yields were somewhat lower.

**Table 2:** Reaction of diethyl zinc with dialkyl itaconate (**1**) ^a^.

Entry	1 (R)	Time (h)	Yield (%) ^b^	*cis*/*trans* ^c^
1	1a (Me)	2	85	62:38
2	1b (Et)	2	71	73:27
3	1c (*n*-Bu)	15	79	86:14
4	1d (*i*-Pr)	18	72	72:28 ^d^

^a^ **1** 1.6 mmol, **1**:Et_2_Zn:CuF(PPh_3_)_3_·2MeOH:Xantphos = 1.0:0.75:1.0%:1.0% (molar ratio); ^b^ isolated yield; ^c^ determined by GC analysis unless noted; ^d^ determined by ^1^H-NMR.

**Scheme 3:**
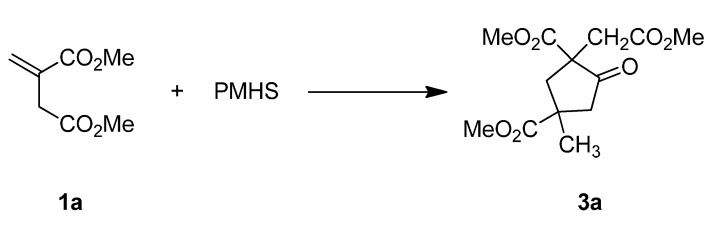
A domino reaction between dimethyl itaconate and PMHS.

Using poly(methylhydrosiloxane) (PMHS) as a hydride source, copper-catalyzed reductive dimerization of **1a** gave 2,4-bis(methoxycarbonyl)-2-(2-methoxy-2-oxoethyl)-4-methylcyclopentanone (**3a**) in a 41% yield (Scheme 3).

## 3. Experimental Section

### 3.1. General Information

All reagents were purchased from Alfa Aesar China (Shanghai, China), and the solvents were bought from Sinoreagent (Shanghai, China). GC analyses were performed on a Shimadzu GC-2010 Gas Chromatograph (Shimadzu Corporation, Kyoto, Japan) using an SE-54 capillary column (30 m × 0.32 mm × 0.4 μm). Mass spectra were recorded in electron impact mode on a HP 6890/5973 GC-MS mass spectrometer (Agilent Technologies, Santa Clara, CA, USA) at 70 eV. High_resolution mass (HRMS) analyses were performed on a Shimadzu LCMS-2020 (Shimadzu Corporation, Kyoto, Japan). Infrared spectra were recorded on a Nicolet 550 FT-IR spectrometer (Thermo Fisher Scientific Inc., Waltham, MA, USA) from 4000 cm^−1^ to 400 cm^−1^, and NMR spectra were recorded on a Bruker Adv. 500 NMR spectrometer (Bruker Inc., Billerica, MA, USA) in CDCl_3_ at 500 MHz and 125 MHz for ^1^H-NMR and ^13^C-NMR, respectively, chemical shifts are given in parts per million (ppm).

### 3.2. General Procedure for the Reactions

Under a nitrogen atmosphere and stirring, a diethyl zinc solution in hexane (1 mol/L, 1.2 mL, 1.2 mmol) was added to a dried Schlenk tube charged with Xantphos (9.3 mg, 0.016 mmol), [CuF(PPh_3_)_3_]·2MeOH (14.9 mg, 0.016 mmol), **1a** (0.253 g, 1.6 mmol) and toluene (2 mL). The mixture was stirred until the **1a** was consumed, as monitored by GC analysis. A saturated ammonium chloride solution in water (2 mL) was added to the mixture to quench the excess diethyl zinc, and then dilute HCl was added to dissolve the solid. The mixture was separated by phase separation, and the aqueous solution was extracted with dichloromethane (10 mL × 3). The combined organic phase was dried and analyzed by GC to determine the diastereomeric ratio. Concentration of the dried organic solution and column chromatography gave **2a** as a colorless oil (0.214 g, 85% yield). Pure *cis*-**2a** and *trans*-**2a** could be obtained by further column chromatography on silica gel using petroleum-ethyl acetate eluent.

*trans-2,4-Bis(methoxycarbonyl)-2-(2-methoxy-2-oxoethyl)-4-propylcyclopentanone* (*trans*-**2a**): Colorless oil, R_f_ = 0.44 (20% EA in PE). ^1^H-NMR (CDCl_3_) δ 3.72 (s, 3H), 3.71 (s, 3H), 3.67 (s, 3H), 3.13 (d, *J* = 17.4 Hz, 1H), 3.00 (dd, *J* = 17.9, 1.3 Hz, 1H), 2.85 (d, *J* = 14.8 Hz, 1H), 2.66 (dd, *J* = 14.8, 1.4 Hz, 1H), 2.54 (d, *J* = 17.5 Hz, 1H), 2.46 (d, *J* = 17.9 Hz, 1H), 1.93–1.84 (m, 1H), 1.75–1.67 (m, 1H), 1.31–1.23 (m, 2H), 0.93 (t, *J* = 7.3 Hz, 3H). ^13^C-NMR (CDCl_3_) δ 209.76, 176.59, 171.15, 170.45, 58.24, 53.18, 52.40, 51.93, 47.83, 46.92, 42.03, 41.82, 38.53, 18.96, 14.28. LRMS *m*/*z* (%) 314 (4, [M]^+^), 282 (31), 272 (1), 255 (8), 244 (6), 223 (37), 207 (3), 195 (17), 172 (3), 156 (100), 127 (92), 113 (5), 99 (9), 85 (1), 71 (2), 55 (4). FAB-HRMS: *m*/*z* calcd for [C_15_H_22_O_7_ + H]^+^ 315.1438, found 315.1440. IR (CH_2_Cl_2_): 2956, 1731, 1265, 733 cm^−1^.

*cis-2,4-Bis(methoxycarbonyl)-2-(2-methoxy-2-oxoethyl)-4-propylcyclopentanone* (*cis*-**2a**): Colorless oil, R_f_ = 0.39 (20% EA in PE). ^1^H-NMR (CDCl_3_) δ 3.72 (s, 3H), 3.66 (s, 3H), 3.64 (s, 3H), 3.31 (dd, *J* = 19.1, 1.8 Hz, 1H), 3.20 (dd, *J* = 14.2, 1.7 Hz, 1H), 2.96 (d, *J* = 17.6 Hz, 1H), 2.91 (d, *J* = 17.6 Hz, 1H), 2.40 (d, *J* = 19.1 Hz, 1H), 2.08 (d, *J* = 14.2 Hz, 1H), 1.89 (ddd, *J* = 13.3, 11.9, 5.2 Hz, 1H), 1.58–1.52 (m, 1H), 1.35–1.16 (m, 2H), 0.90 (t, *J* = 7.3 Hz, 3H). ^13^C-NMR (CDCl_3_) δ 211.39, 176.13, 171.20, 170.29, 57.95, 52.94, 52.43, 52.09, 48.92, 46.29, 43.82, 42.99, 38.86, 19.25, 14.36. LRMS *m*/*z* (%) 314 (3, [M]^+^), 282 (43), 272 (1), 255 (8), 244 (9), 223 (43), 207 (1), 195 (21), 172 (3), 156 (100), 127 (98), 113 (9), 99 (14), 85 (2), 71 (2), 55 (4). FAB-HRMS: *m*/*z* calcd for [C_15_H_22_O_7_ + H]^+^ 315.1438, found 315.1442. IR (CH_2_Cl_2_): 2923, 1732, 1265, 737 cm^−1^.

*trans****-****2,4-Bis(ethoxycarbonyl)-2-(2-ethoxy-2-oxoethyl)-4-propylcyclopentanone* (*trans*-**2b**): Colorless oil, R_f_ = 0.54 (15% EA in PE). ^1^H-NMR (CDCl_3_) δ 4.26–4.05 (m, 6H), 3.13 (d, *J* = 17.5 Hz, 1H), 2.98 (d, *J* = 17.0 Hz, 1H), 2.85 (d, *J* = 14.8 Hz, 1H), 2.66 (dd, *J* = 14.7, 0.8 Hz, 1H), 2.51 (d, *J* = 17.5 Hz, 1H), 2.44 (d, *J* = 17.9 Hz, 1H), 1.90 (ddd, *J* = 13.2, 11.0, 5.8 Hz, 1H), 1.70 (ddd, *J* = 13.4, 10.8, 5.9 Hz, 1H), 1.33–1.20 (m, 11H), 0.93 (t, *J* = 7.3 Hz, 3H). ^13^C-NMR (CDCl_3_) δ 210.14, 176.22, 170.79, 170.05, 62.20, 61.36, 60.92, 58.41, 47.76, 46.99, 42.00, 41.95, 38.86, 18.99, 14.42, 14.19, 14.18, 14.01. LRMS *m*/*z* (%) 356 (5, [M]^+^), 310 (24), 283 (6), 268 (5), 237 (30), 209 (17), 187 (4), 170 (100), 141 (97), 127 (2), 113 (24), 99 (2), 85 (6), 55 (4). FAB-HRMS: *m*/*z* calcd for [C_18_H_28_O_7_ + H]^+^ 357.1908, found 357.1909. IR (CDCl_3_): 2963, 1727, 1186, 905, 729 cm^−1^.

*cis**-**2,4-Bis(ethoxycarbonyl)-2-(2-ethoxy-2-oxoethyl)-4-propylcyclopentanone* (*cis*-**2b**): Colorless oil, R_f_ = 0.48 (15% EA in PE). ^1^H-NMR (CDCl_3_) δ 4.21–4.09 (m, 5H), 4.07–4.00 (m, 1H), 3.31 (dd, *J* = 19.1, 1.0 Hz, 1H), 3.22 (d, *J* = 14.2 Hz, 1H), 2.95 (d, *J* = 17.6 Hz, 1H), 2.89 (d, *J* = 17.6 Hz, 1H), 2.39 (d, *J* = 19.1 Hz, 1H), 2.07 (d, *J* = 14.2 Hz, 1H), 1.94–1.84 (m, 1H), 1.64–1.51 (m, 1H), 1.28 (t, *J* = 7.2 Hz, 3H), 1.27–1.23 (m, 5H), 1.20 (t, *J* = 7.1 Hz, 3H), 0.90 (t, *J* = 7.3 Hz, 3H). ^13^C-NMR (CDCl_3_) δ 211.79, 175.65, 170.81, 169.87, 62.08, 61.28, 61.10, 58.08, 48.63, 46.21, 43.94, 42.90, 39.16, 19.17, 14.42, 14.30, 14.25, 13.97. LRMS *m*/*z* (%) 356 (5, [M]^+^), 310 (25), 283 (8), 268 (6), 255 (2), 237 (30), 209 (20), 187 (5), 170 (100), 141(99), 127 (3), 113 (27), 99 (3), 85 (6), 71 (2), 55 (5). FAB-HRMS: *m*/*z* calcd for [C_18_H_28_O_7_ + H]^+^ 357.1908, found 357.1910. IR (CDCl_3_): 2961, 1728, 1180, 1030, 907, 730 cm^−1^.

*trans****-****2,4-Bis(butoxycarbonyl)-2-(2-butoxy-2-oxoethyl)-4-propylcyclopentanone* (*trans*-**2c**): Colorless oil, R_f_ = 0.54 (10% EA in PE). ^1^H-NMR (CDCl_3_) δ 4.17–3.98 (m, 6H), 3.14 (d, *J* = 17.5 Hz, 1H), 2.97 (d, *J* = 17.7 Hz, 1H), 2.86 (d, *J* = 14.8 Hz, 1H), 2.65 (dd, *J* = 14.7, 0.8 Hz, 1H), 2.50 (d, *J* = 17.5 Hz, 1H), 2.43 (d, *J* = 17.8 Hz, 1H), 1.90 (ddd, *J* = 13.4, 10.5, 6.3 Hz, 1H), 1.70 (ddd, *J* = 13.4, 10.3, 6.5 Hz, 1H), 1.64–1.53 (m, 6H), 1.40–1.32 (m, 6H), 1.30–1.25 (m, 2H), 0.99–0.88 (m, 12H). ^13^C-NMR (CDCl_3_) δ 210.16, 176.41, 170.95, 170.19, 66.14, 65.33, 64.90, 58.53, 47.95, 47.06, 42.14, 42.11, 38.90, 30.68, 30.66, 30.55, 19.24, 19.21, 19.17, 19.12, 14.47, 13.81, 13.78 (2C). LRMS *m*/*z* (%) 440 (2, [M]^+^), 368 (10), 339 (3), 324 (3), 310 (5), 265 (6), 243 (3), 210 (10), 198 (47), 181 (6), 169 (6), 142 (100), 113 (26), 99 (1), 85 (2), 55 (3). FAB-HRMS: *m*/*z* calcd for [C_24_H_40_O_7_ + H]^+^ 441.2847, found 441.2847. IR (CH_2_Cl_2_): 2960, 1730, 1183, 909, 731 cm^−1^.

*cis**-**2,4-Bis(butoxycarbonyl)-2-(2-butoxy-2-oxoethyl)-4-propylcyclopentanone* (*cis*-**2c**): Colorless oil, R_f_ = 0.48 (10% EA in PE). ^1^H-NMR (CDCl_3_) δ 4.09 (t, *J* = 6.6 Hz, 3H), 4.05 (t, *J* = 6.7 Hz, 2H), 3.97 (dt, *J* = 11.1, 6.7 Hz, 1H), 3.30 (d, *J* = 19.0 Hz, 1H), 3.22 (d, *J* = 14.2 Hz, 1H), 2.96 (d, *J* = 17.6 Hz, 1H), 2.88 (d, *J* = 17.6 Hz, 1H), 2.38 (d, *J* = 19.0 Hz, 1H), 2.06 (d, *J* = 14.2 Hz, 1H), 1.89 (td, *J* = 12.4, 4.9 Hz, 1H), 1.68–1.51 (m, 7H), 1.43–1.19 (m, 8H), 1.09–0.70 (m, 12H). ^13^C-NMR (CDCl_3_) δ 211.59, 175.65, 170.87, 169.87, 65.94, 65.22, 64.99, 58.13, 48.70, 46.18, 43.92, 42.95, 39.18, 30.69, 30.66, 30.45, 19.33, 19.19, 19.06, 14.38, 13.83, 13.78. LRMS *m*/*z* (%) 440 (2, [M]^+^, 368 (10), 339 (2), 324 (3), 310 (5), 265 (3), 243 (6), 210 (4), 198 (48), 181 (10), 169 (11), 142 (100), 113 (25), 99 (1), 85 (2), 55 (3). IR (CH_2_Cl_2_): 2959, 1758, 1176 cm^−1^.

*trans****-****2,4-Bis(isopropoxycarbonyl)-2-(2-isopropoxy-2-oxoethyl)-4-propylcyclopentanone* (*trans*-**2d**): Colorless oil, R_f_ = 0.44 (15% EA in PE). ^1^H-NMR (CDCl_3_) δ 5.04–4.92 (m, 3H), 3.09 (d, *J* = 17.5 Hz, 1H), 2.95 (d, *J* = 17.8 Hz, 1H), 2.83 (d, *J* = 14.8 Hz, 1H), 2.63 (d, *J* = 14.7 Hz, 1H), 2.46 (d, *J* = 17.5 Hz, 1H), 2.41 (d, *J* = 17.8 Hz, 1H), 1.90 (ddd, *J* = 13.3, 10.3, 6.5 Hz, 1H), 1.72–1.64 (m, 1H), 1.31–1.19 (m, 20H), 0.93 (t, *J* = 7.3 Hz, 3H). ^13^C-NMR (CDCl_3_) δ 210.34, 175.80, 170.34, 169.61, 69.84, 68.81, 68.44, 58.59, 47.75, 46.96, 42.01, 42.00, 39.11, 21.90, 21.84, 21.75, 21.74, 21.68, 21.52, 19.02, 14.51. LRMS *m*/*z* (%) 398 (1, [M]^+^), 355 (5), 339 (4), 311 (8), 297 (19), 271 (3), 237 (9), 215 (38), 184 (31), 173 (30), 155 (11), 142 (100), 113 (38), 99 (4), 85 (5), 71 (1), 55 (4). FAB-HRMS: *m*/*z* calcd for [C_21_H_34_O_7_ + H]^+^: 399.2378, found: 399.2374. IR (CH_2_Cl_2_): 2983, 1723, 1102, 905, 726 cm^−1^.

*cis****-****2,4-Bis(isopropoxycarbonyl)-2-(2-isopropoxy-2-oxoethyl)-4-propylcyclopentanone* (*cis*-**2d**): Colorless oil, R_f_ = 038 (15% EA in PE). ^1^H-NMR (CDCl_3_) δ 5.06–4.89 (m, 3H), 3.28 (d, *J* = 19.0 Hz, 1H), 3.21 (d, *J* = 14.4 Hz, 1H), 2.92 (d, *J* = 17.4 Hz, 1H), 2.81 (d, *J* = 17.4 Hz, 1H), 2.39–2.32 (m, 1H), 2.03 (d, *J* = 14.2 Hz, 1H), 1.92–1.84 (m, 1H), 1.57–1.49 (m, 1H), 1.32–1.15 (m, 23H). ^13^C-NMR (CDCl_3_) δ 211.80, 175.03, 170.34, 169.29, 69.79, 68.67, 68.61, 58.36, 48.29, 46.01, 43.90, 42.83, 39.53, 21.93, 21.89, 21.86, 21.83, 21.53, 21.51, 19.06, 14.46. LRMS *m*/*z* (%) 398 (2, [M]^+^), 355 (5), 339 (3), 311 (2), 297 (27), 271 (2), 237 (16), 215 (39), 184 (29), 173 (29), 155 (11), 142 (100), 113 (31), 99 (3), 85 (3), 71 (1), 55 (2). FAB-HRMS: *m*/*z* calcd for [C_21_H_34_O_7_ + H]^+^ 399.2378, found: 399.2378. IR (CH_2_Cl_2_): 2982, 1724, 1265, 1105, 735 cm^−1^.

#### Reductive Dimerization of **1a**

Under a nitrogen atmosphere and stirring, PMHS (0.12 mL, 2.0 mmol SiH) was added to a dried Schlenk tube charged with DPEphos (18.9 mg, 0.035 mmol), [CuF(PPh_3_)_3_]·2MeOH (28.0 mg, 0.030 mmol), **1a** (0.205 g, 1.30 mmol) and toluene (2.0 mL). The mixture was stirred until **1a** was consumption as monitored by TLC. A saturated ammonium fluoride solution in water (2 mL) was added to the mixture to quench the reaction. After stirred for 0.5 h, the mixture was separated by phase separation, and the aqueous solution was extracted with dichloromethane (3 mL × 10 mL). The combined organic phase was dried and analyzed by GC to determine the diastereomeric ratio. Concentration of the dried organic solution and column chromatography gave **3a** as a colorless oil (0.076 g, 41% yield). Pure diastereomers of **3a** could be obtained by further column separation using petroleum ether-ethyl acetate eluent.

*trans*-*2,4-Bis(methoxycarbonyl)-2-(2-methoxy-2-oxoethyl)-4-methylcyclopentanone* (*trans*-**3a)**: R_f_ = 0.39 (20% EA in PE). ^1^H-NMR (CDCl_3_) δ 3.61 (s, 3H), 3.59 (s, 3H), 3.54 (s, 3H), 3.02 (d, *J* = 17.5 Hz, 1H), 2.85 (d, *J* = 17.8 Hz, 1H),2.69 (d, *J* = 14.8 Hz, 1H), 2.51 (d, *J* = 14.1 Hz, 1H), 2.50 (d, *J* = 17.7 Hz, 1H) 2.35 (d, *J* = 18.0 Hz, 1H), 1.37 (s, 3H). ^13^C-NMR (CDCl_3_) δ 209.48, 176.91, 170.90, 170.25, 58.46, 52.94, 52.28, 51.67, 48.28, 43.01, 42.91, 38.64, 25.13. MS *m*/*z* (%): 286 (3, [M]^+^), 254 (53), 222 (3), 212 (2), 195 (46), 167 (35), 153 (2), 140 (6), 128 (100), 113 (7), 100 (43), 91 (1), 77 (17), 69 (17), 59 (15), 44 (7).

*cis-2,4-Bis(methoxycarbonyl)-2-(2-methoxy-2-oxoethyl)-4-methylcyclopentanone* (*cis*-**3a**): R_f_ = 0.34 (20% EA in PE). ^1^H-NMR (CDCl_3_) δ 3.72 (s, 3H), 3.67 (s, 3H), 3.65 (s, 3H), 3.32 (d, *J* = 18.9 Hz, 1H), 3.24 (d, *J* = 14.2 Hz, 1H), 2.98 (d, *J* = 17.6 Hz, 1H), 2.90 (d, *J* = 17.6 Hz, 1H), 2.39 (d, *J* = 19.0 Hz, 1H), 2.08 (d, *J* = 14.2 Hz, 1H), 1.45 (s, 3H). ^13^C-NMR (CDCl_3_) δ 211.01, 176.61, 170.95, 170.07, 58.27, 52.78, 52.43, 51.91, 47.75, 44.17, 43.44, 38.73, 26.91. MS *m*/*z* (%): 286 (4, [M]^+^), 254 (50), 222 (3), 212 (2), 195 (45), 167 (26), 153 (1), 140 (6), 128 (100), 113 (6), 100 (23), 91 (1), 79 (13), 69 (15), 59 (11), 41 (5).

## 4. Conclusions

As a summary, copper-diphosphorous is efficient in catalyzing the diethyl zinc (or a silane)-induced conjugate addition-dimerization/cyclization domino reaction of itaconates, giving 2,4-bis(alkoxycarbonyl)-2-(2-alkoxy-2-oxoethyl)-4-alkylcyclopentanones in moderate to high yields. The proportion of dominant diastereomer could be varied by using different copper catalysts.

## Supplementary Materials

Supplementary materials can be accessed at: http://www.mdpi.com/1420-3049/20/08/15023/s1.
