# Supplementary material for: Copper-Catalyzed Dimerization/Cyclization of Itaconates
Source: Molecules. 2015 Aug 17;20(8):15023–32. doi: 10.3390/molecules200815023 (PMC6332366; doi:10.3390/molecules200815023)
Supplement: Supplementary file 1 [file molecules-20-15023-s001.pdf]

Supplementary Materials

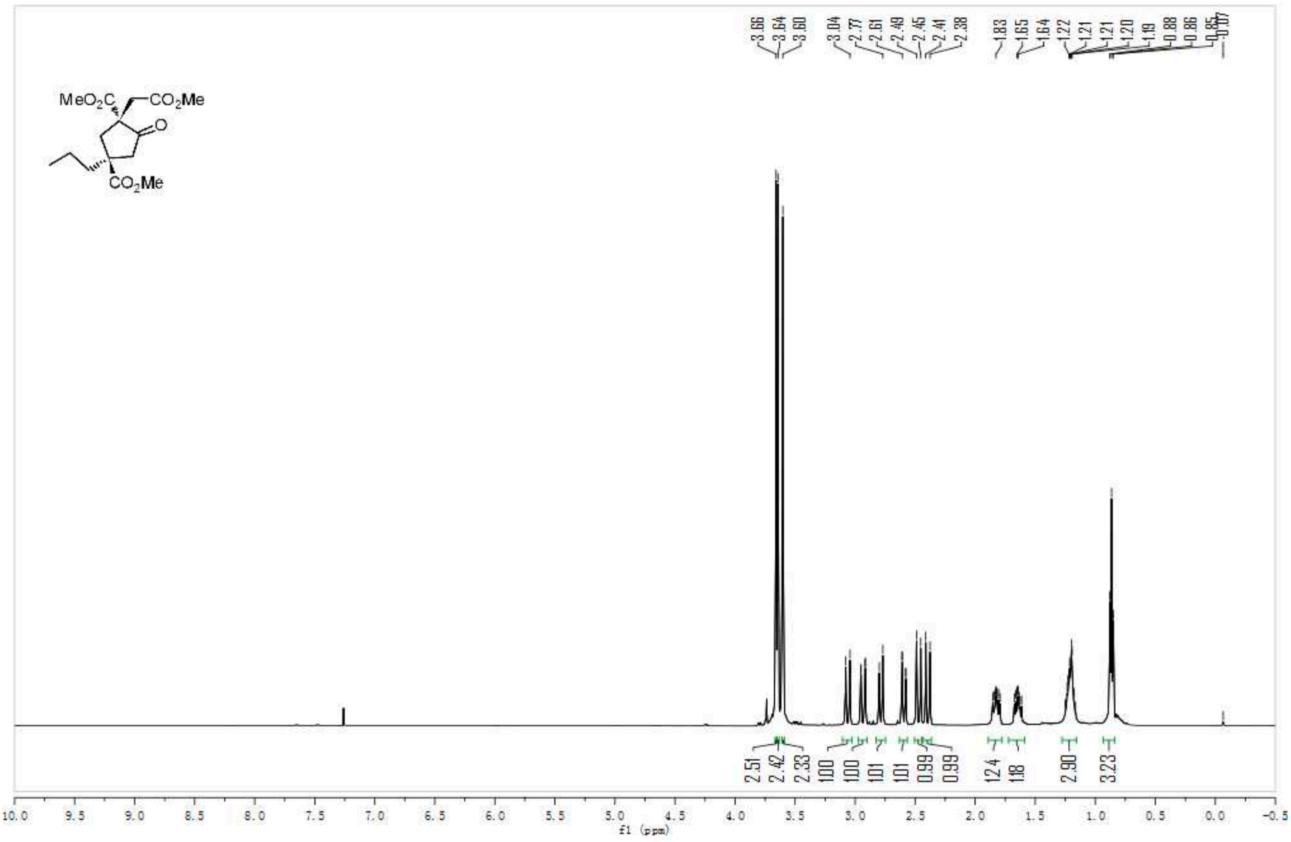

Figure S1. ^{1}H-NMR of *trans*-**2a**.

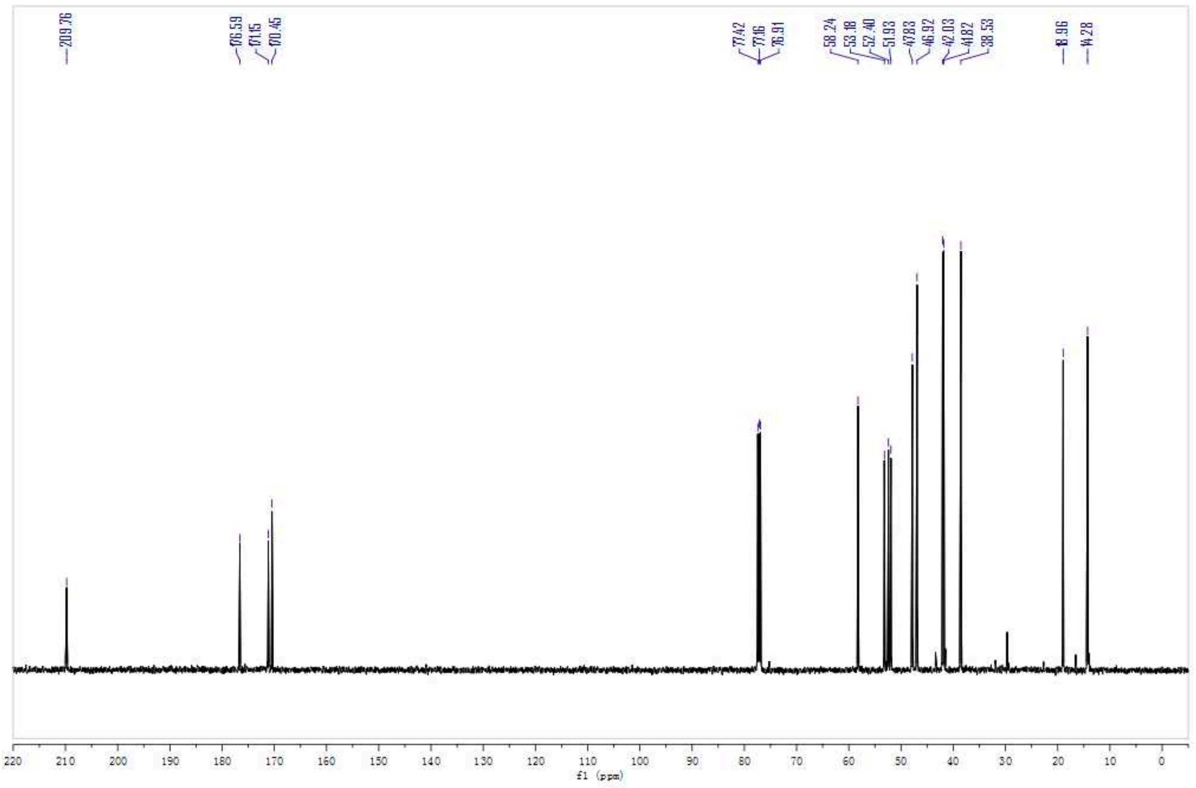

Figure S2. ^{13}C-NMR of *trans*-**2a**.

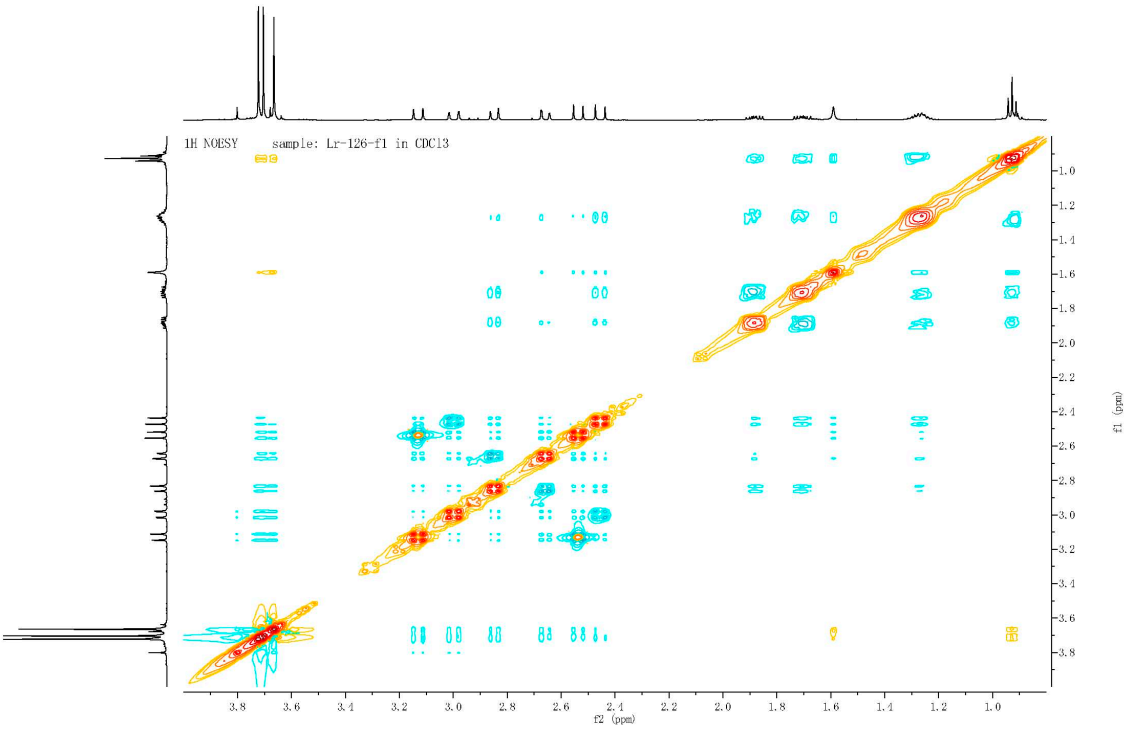

Figure S3. NOESY of *trans*-**2a**.

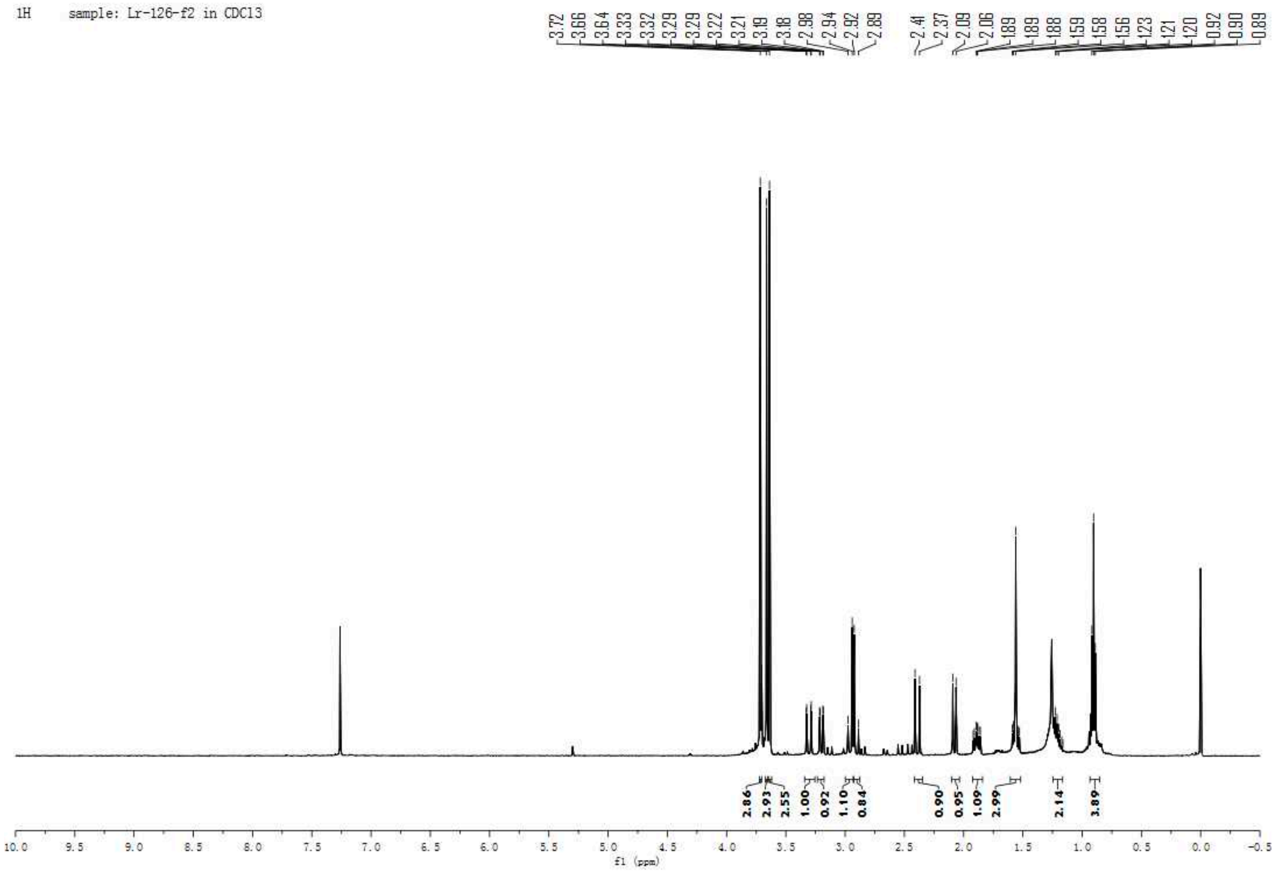

Figure S4. ^{1}H-NMR of *cis*-**2a**.

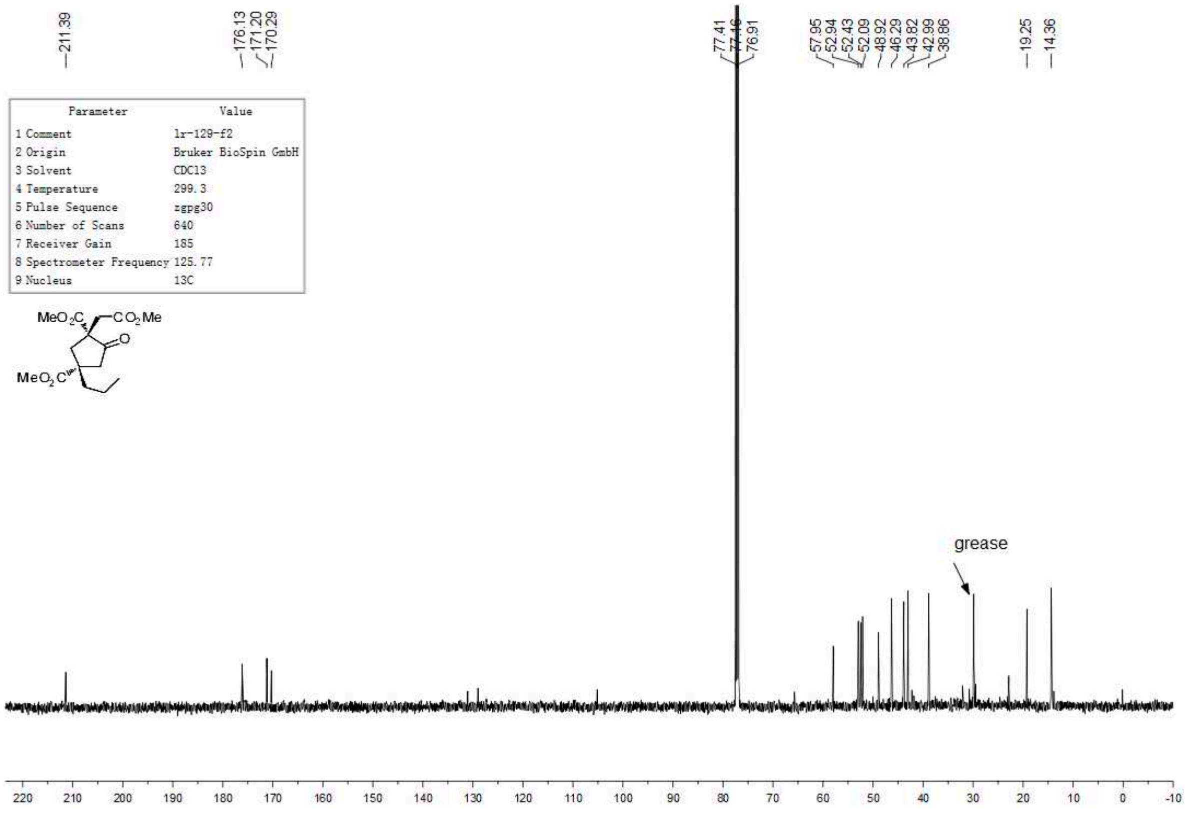

Figure S5. ^{13}C-NMR of *cis*-**2a**.

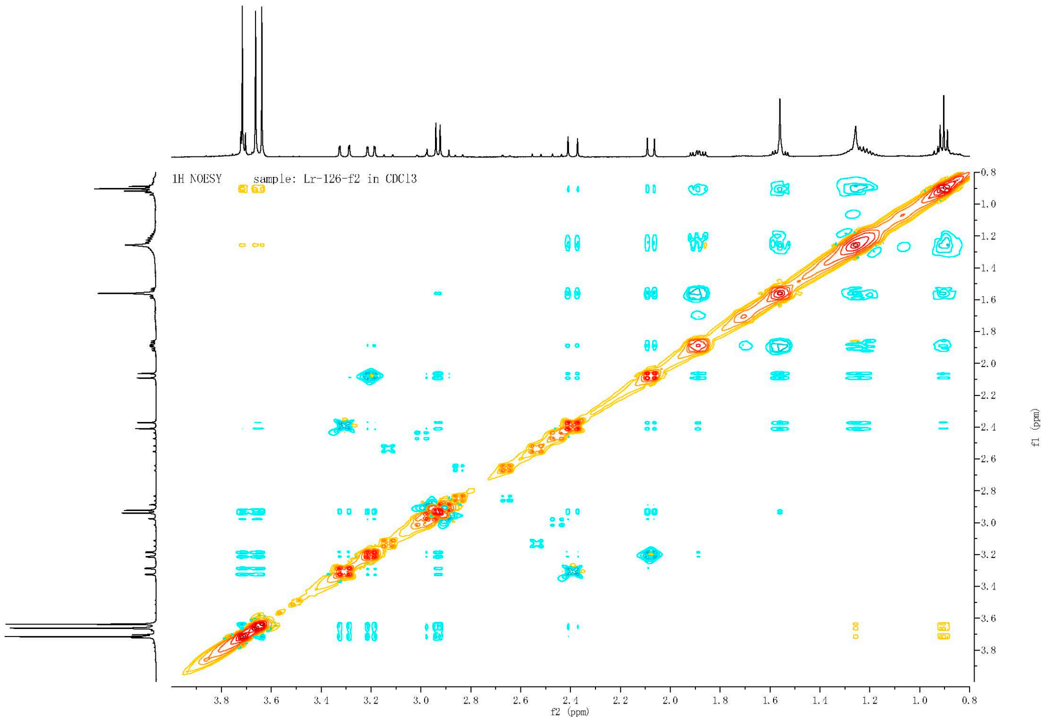

Figure S6. NOESY of *cis*-**2a**.

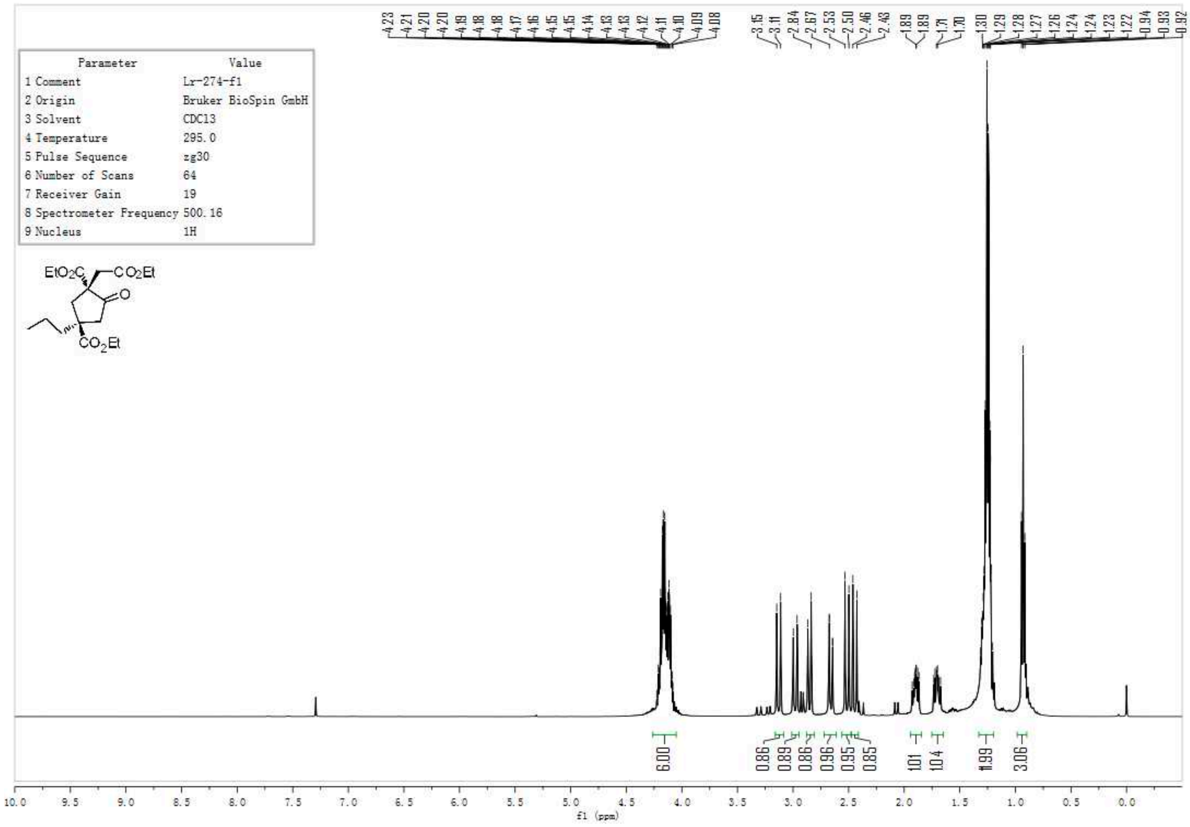

Figure S7. ^{1}H-NMR of *trans*-**2b**.

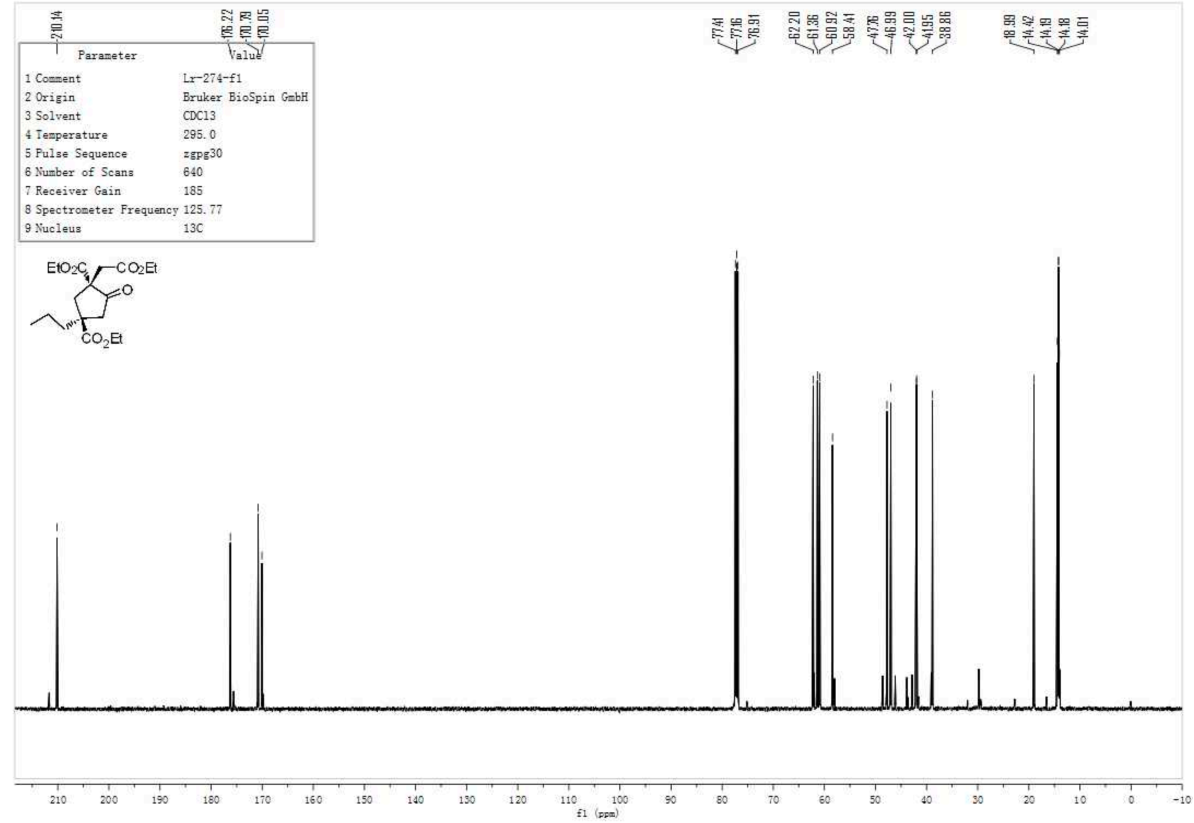

Figure S8. ^{13}C-NMR of *trans*-**2b**.

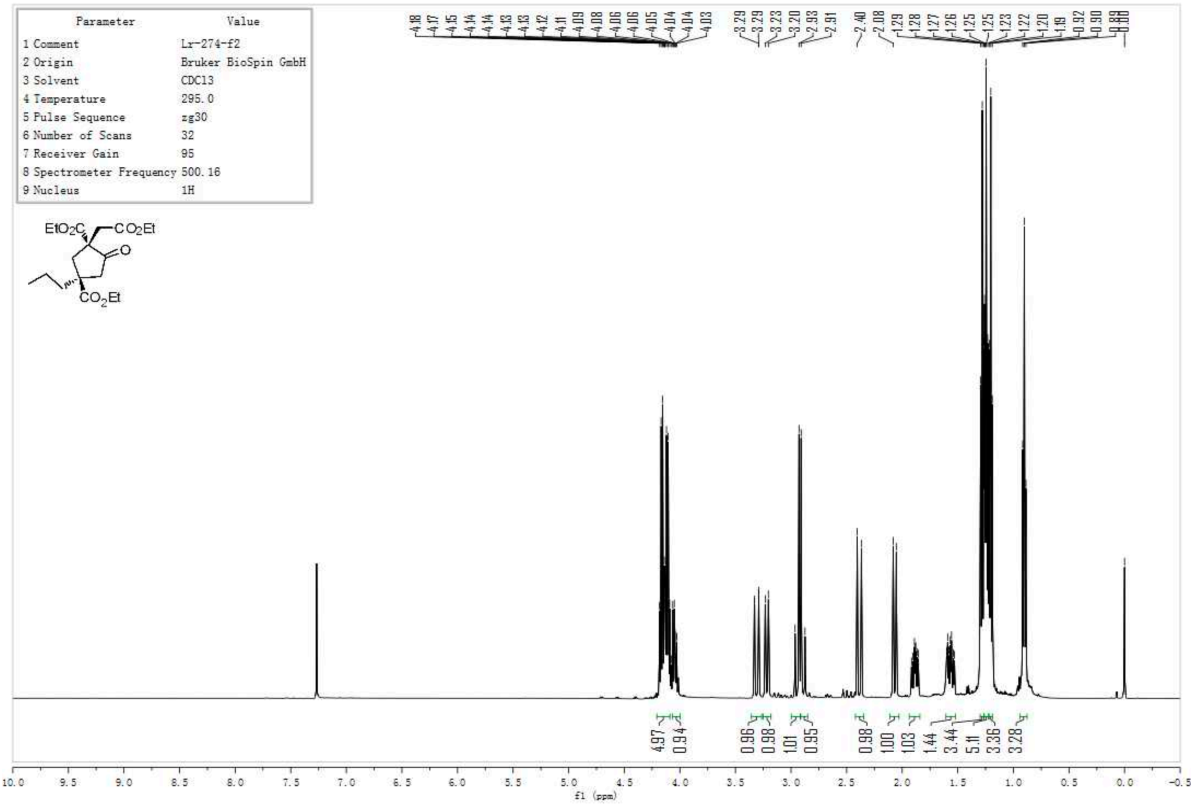

Figure S9. ^{1}H-NMR of *cis*-**2b**.

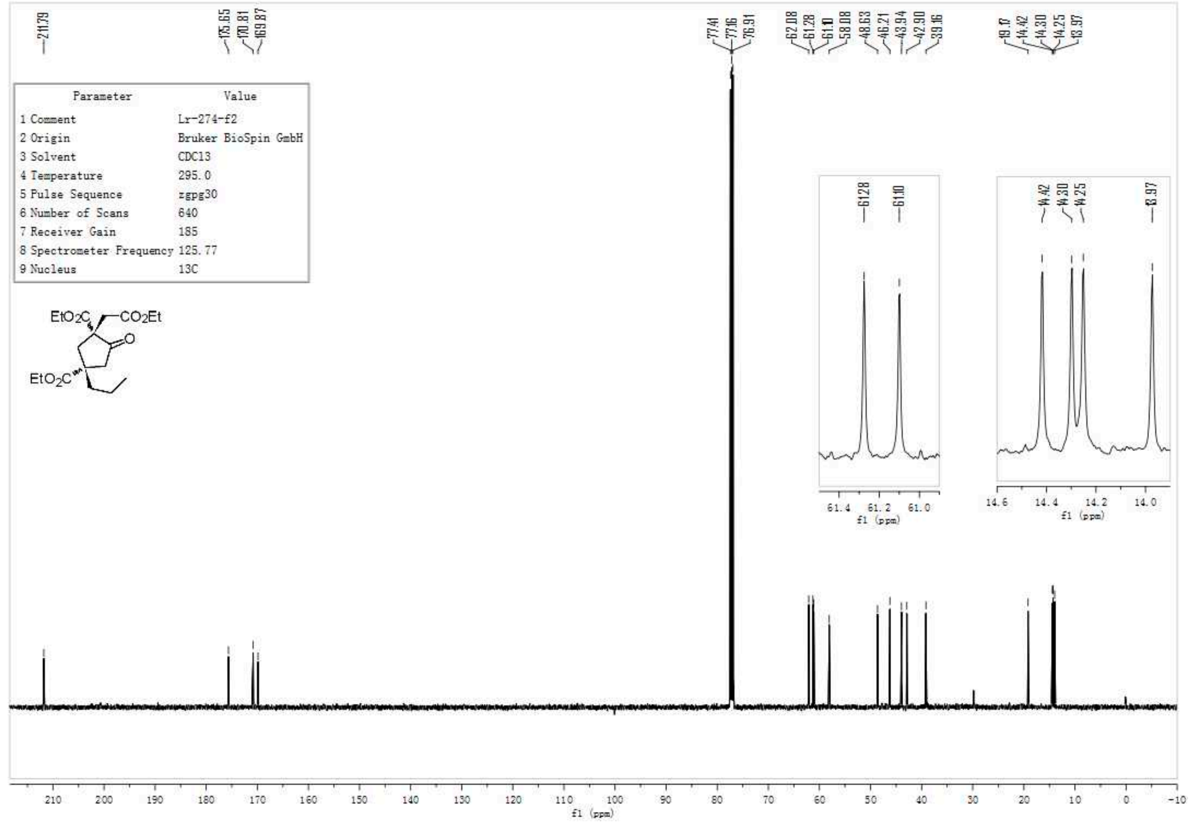

Figure S10. ^{13}C-NMR of *cis*-**2b**.

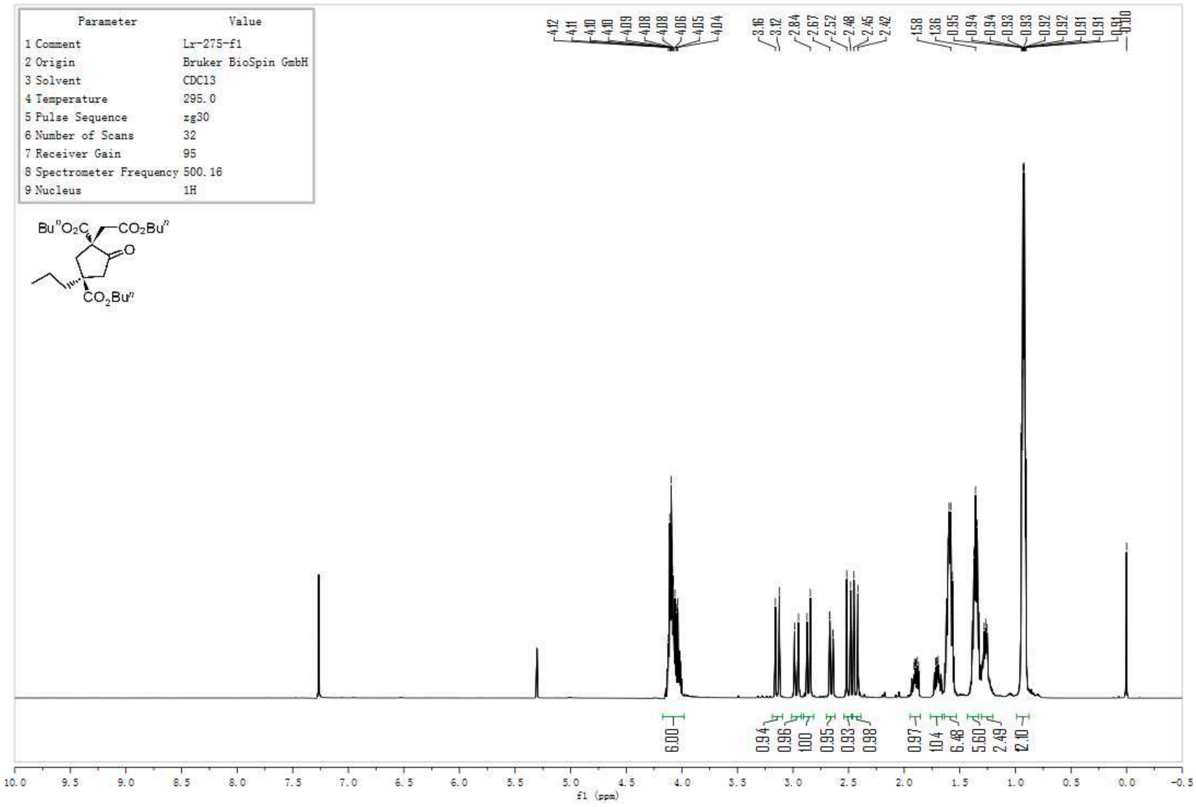

Figure S11. ^{1}H-NMR of *trans*-**2c**.

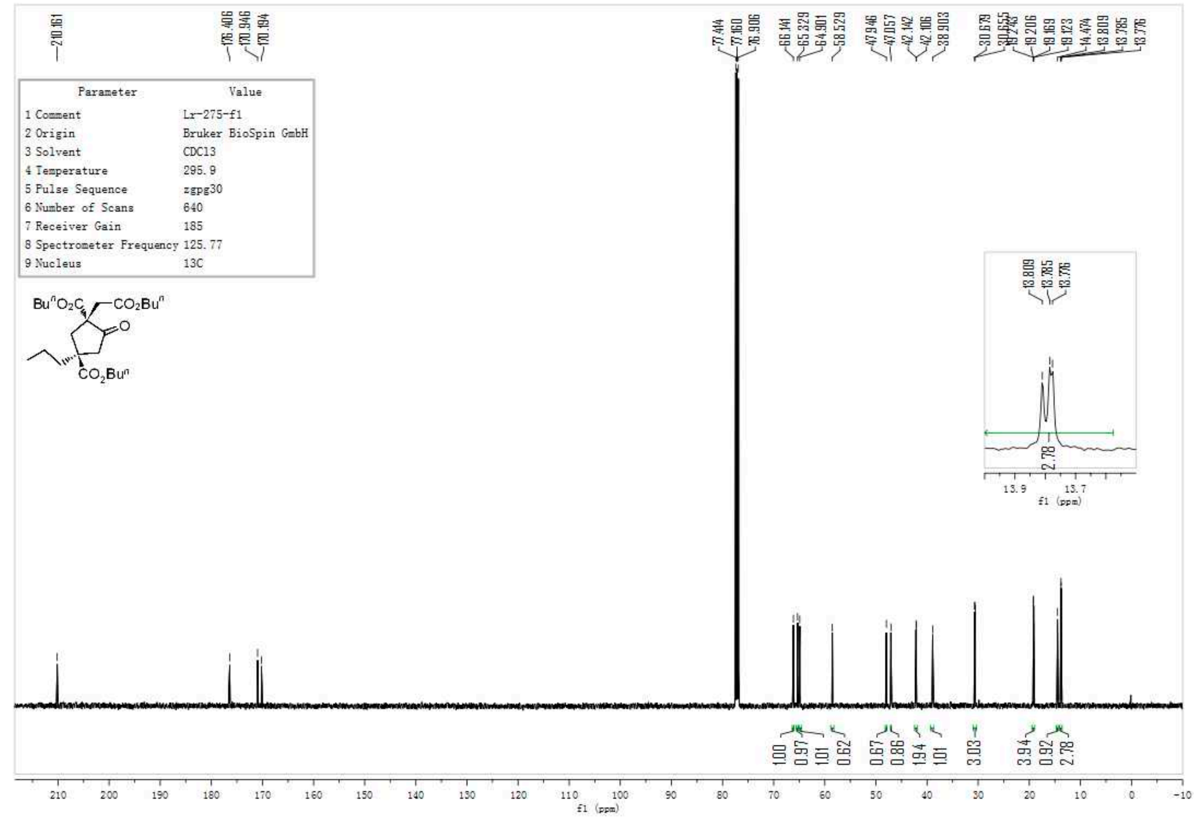

Figure S12. ^{13}C-NMR of *trans*-**2c**.

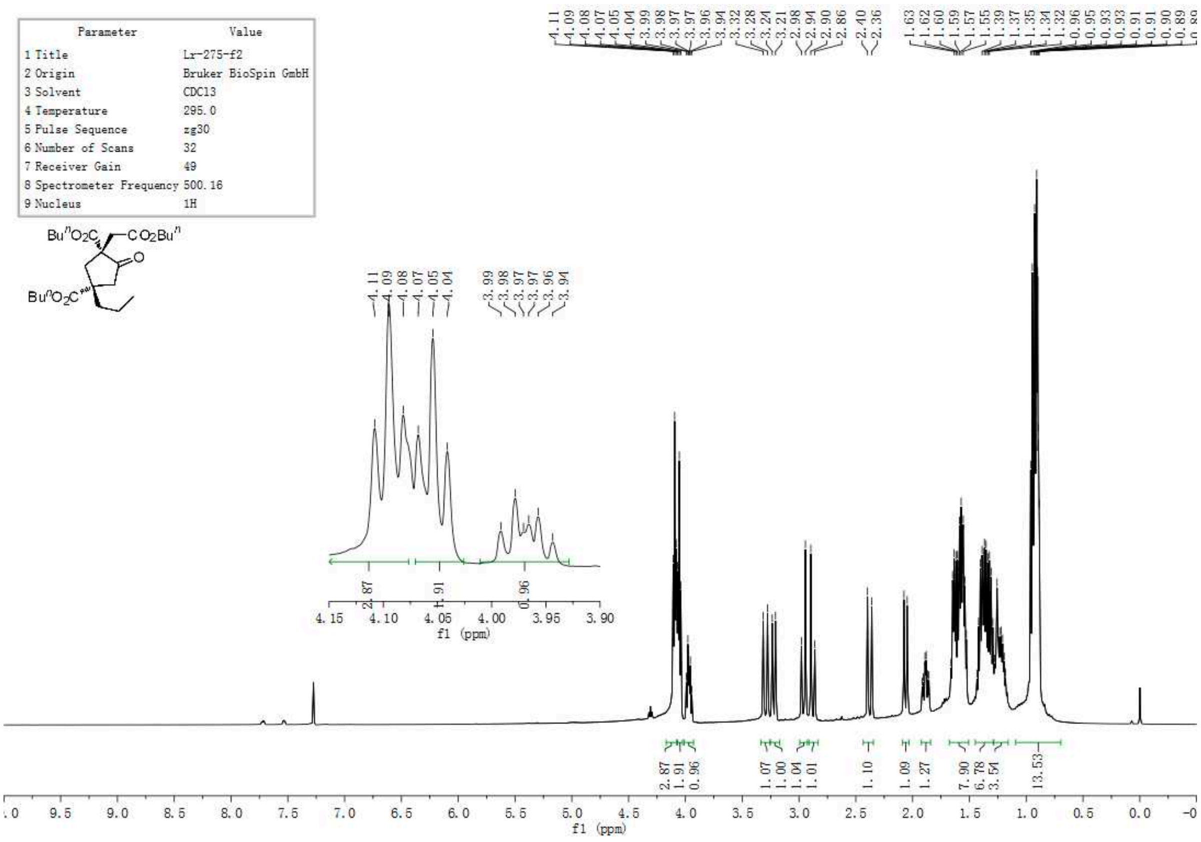

Figure S13. ^{1}H-NMR of *cis*-**2c**.

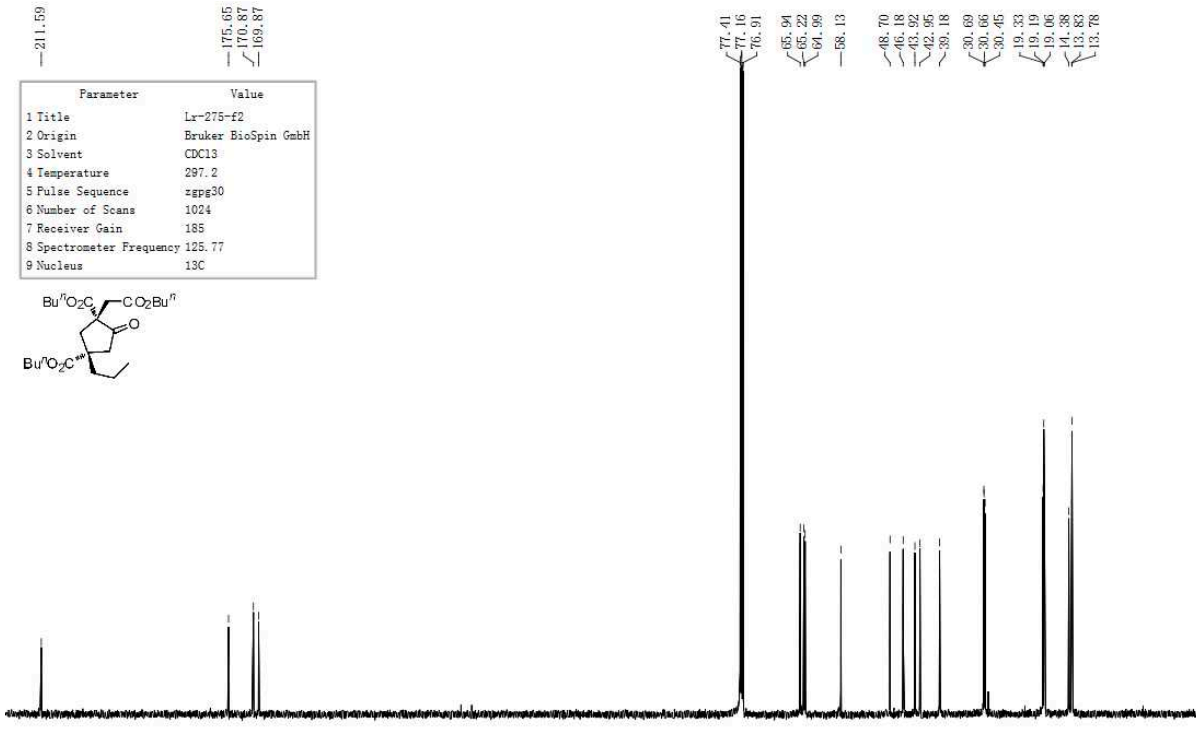

Figure S14. ^{13}C-NMR of *cis*-**2c**.

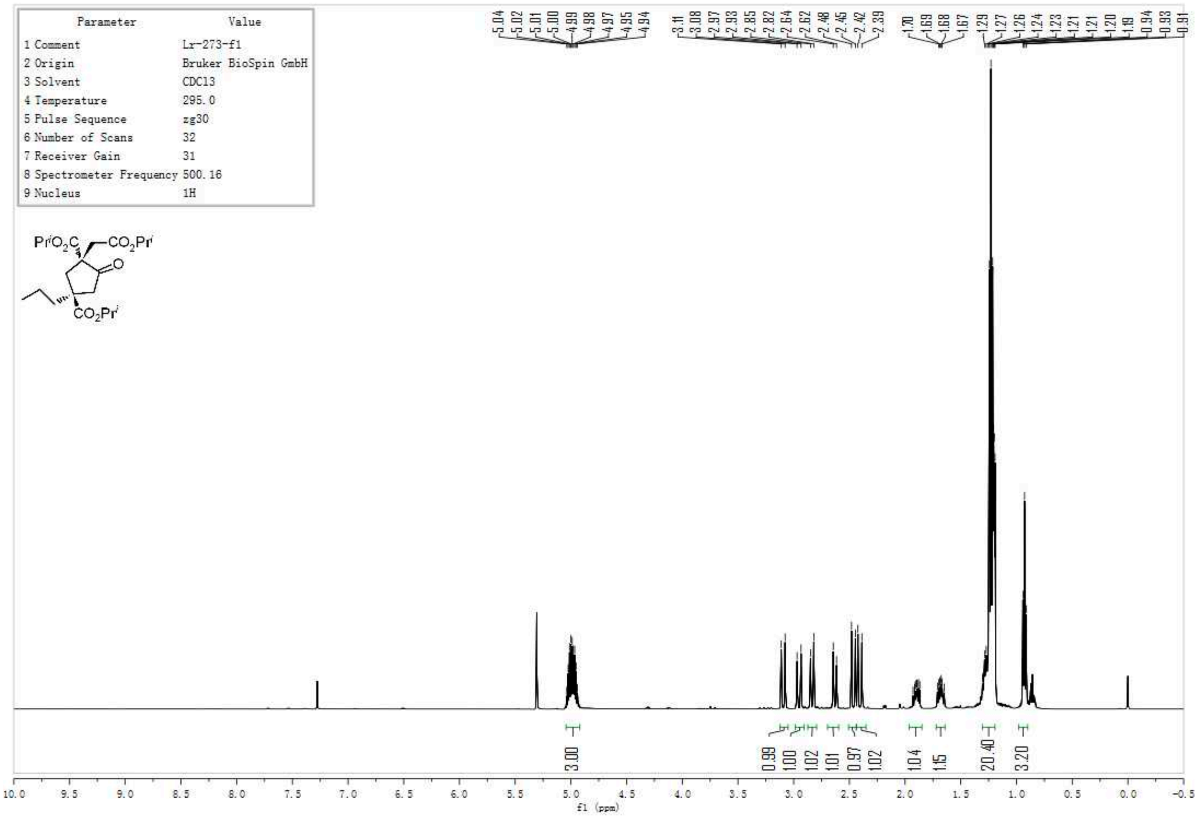

Figure S15. ^{1}H-NMR of *trans*-**2d**.

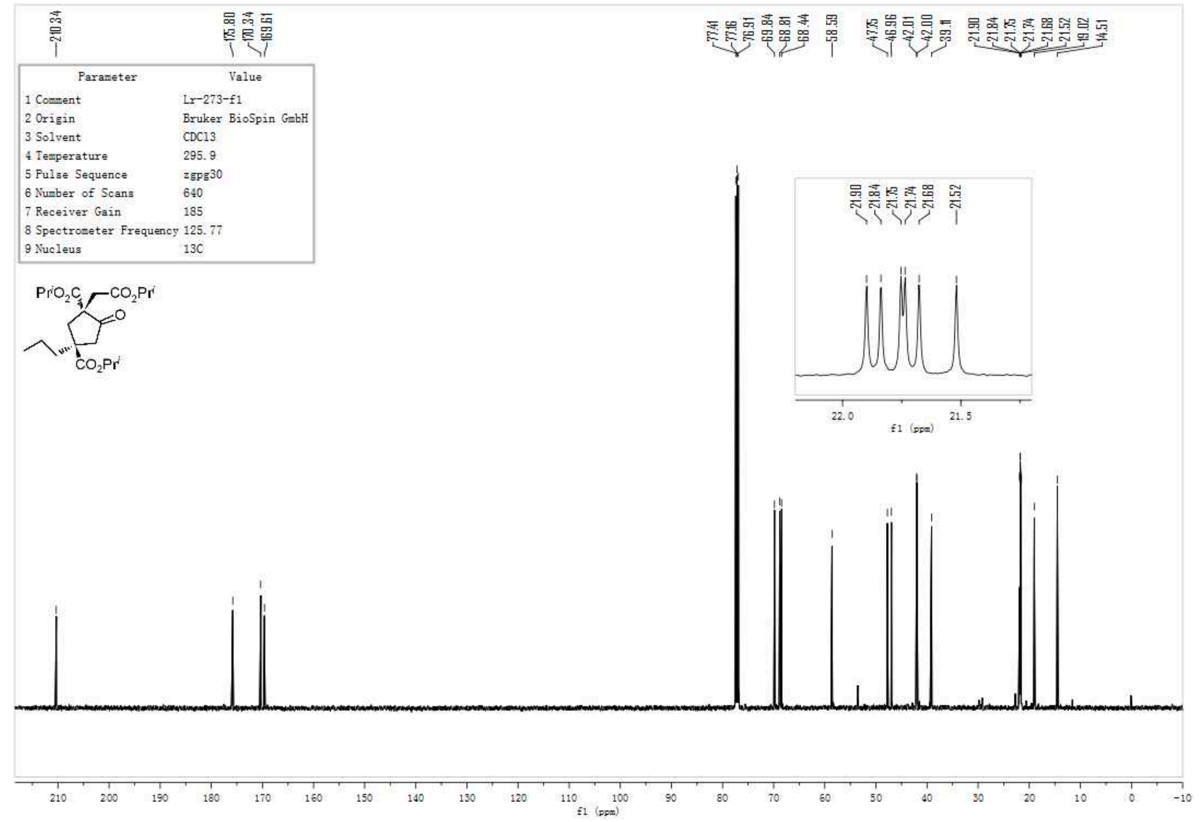

Figure S16. ^{13}C-NMR of *trans*-**2d**.

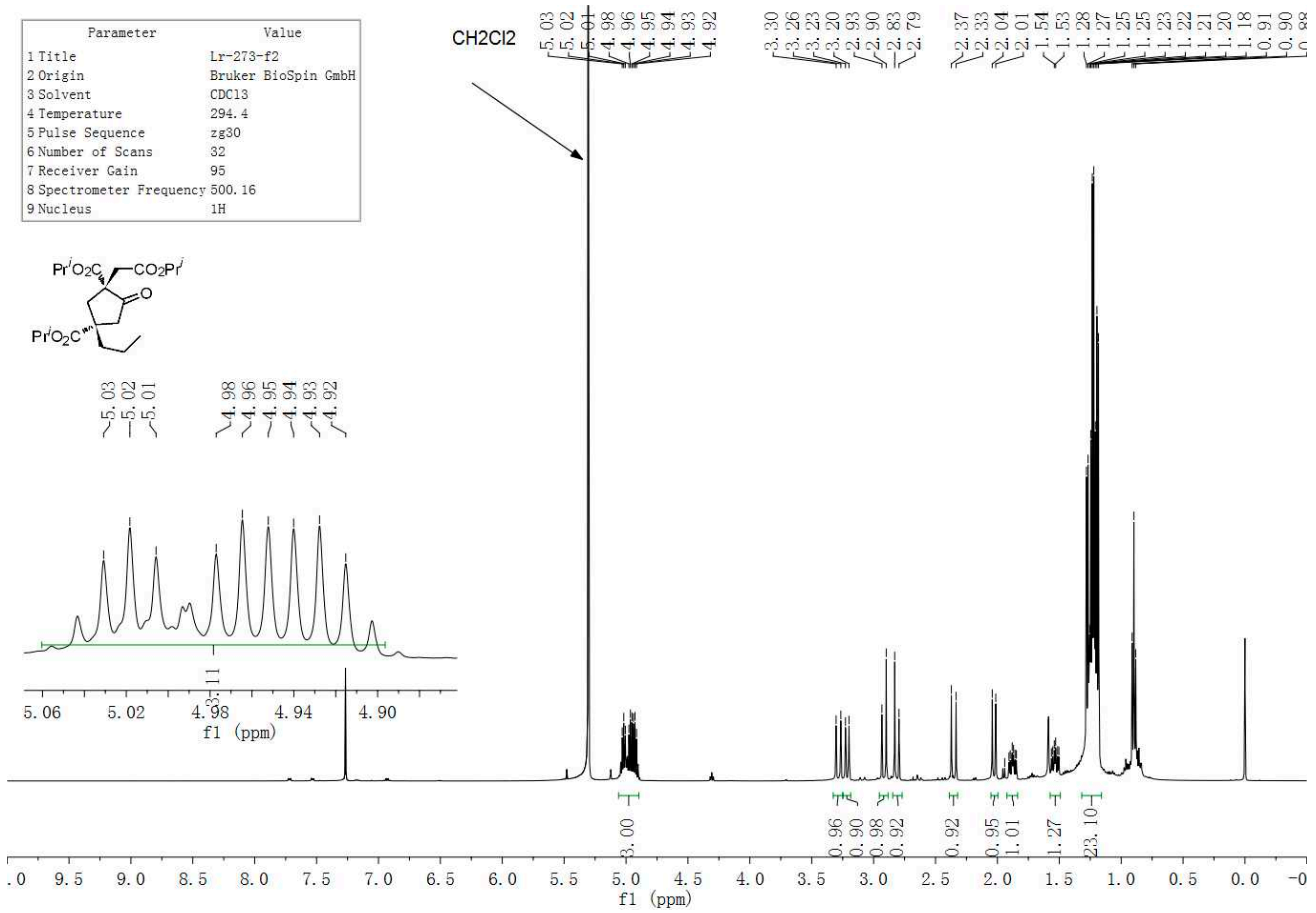

Figure S17. ^{1}H-NMR of *cis*-**2d**.

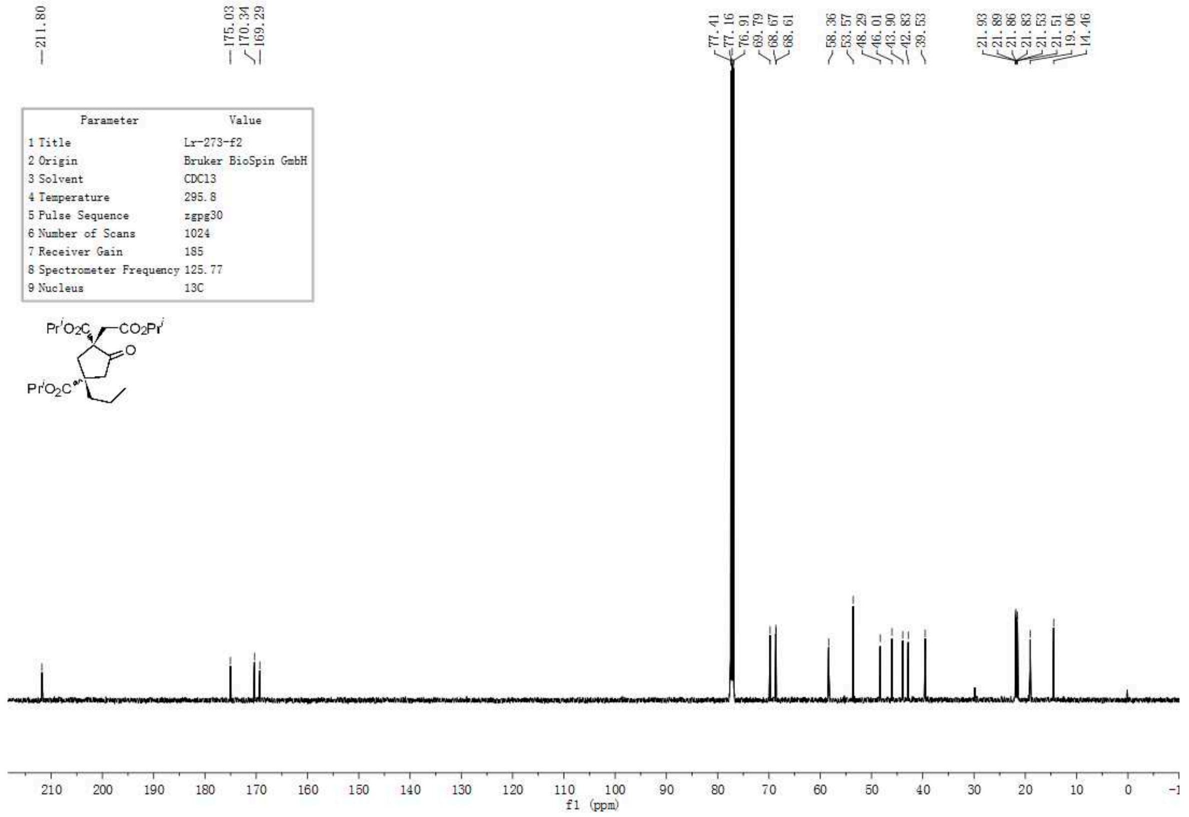

Figure S18. ^{13}C-NMR of *cis*-**2d**.

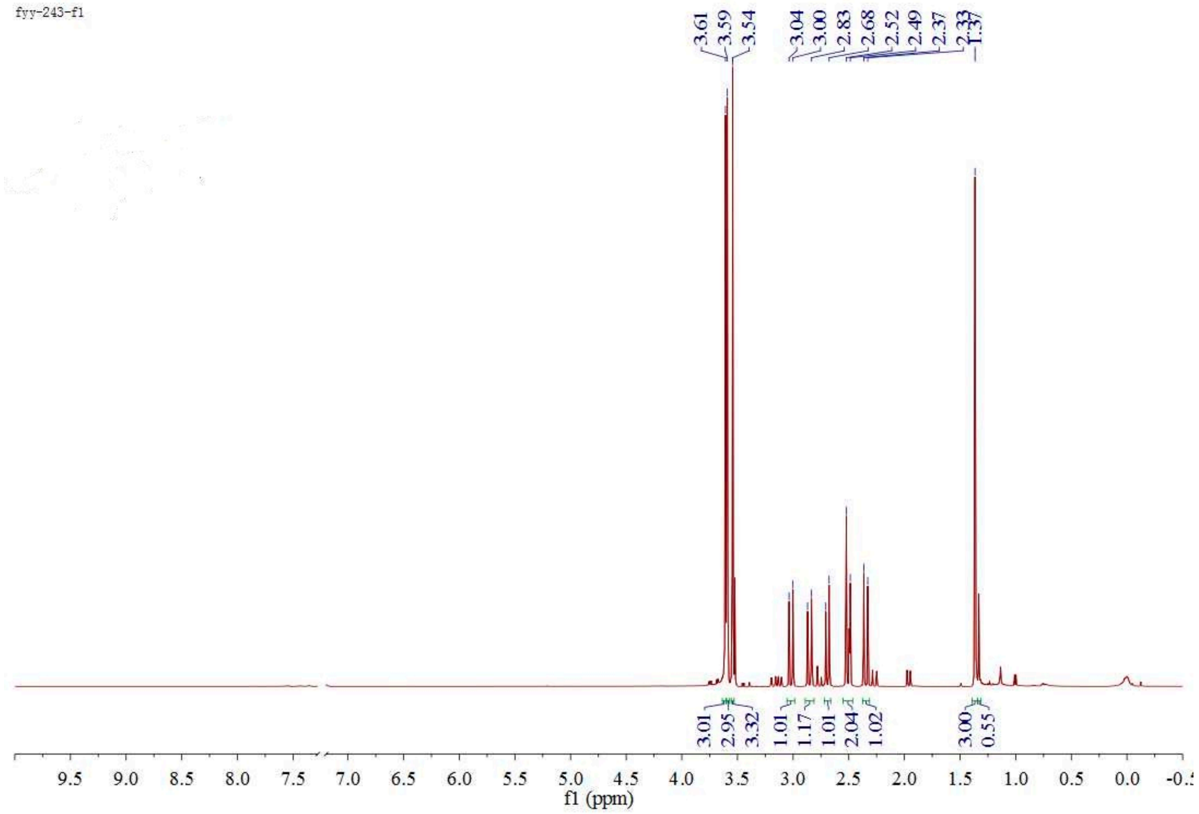

Figure S19. ^{1}H NMR of *cis*-**3a**.

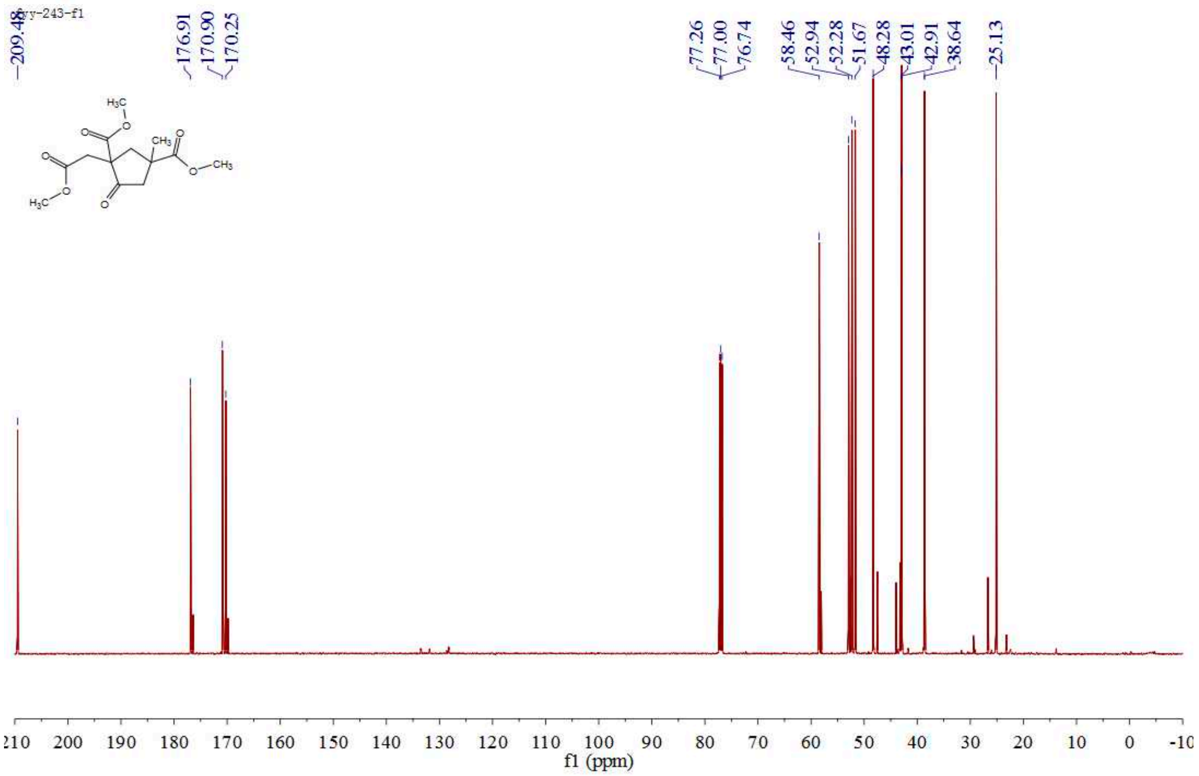

Figure S20. ^{13}C NMR of *cis*-**3a**.

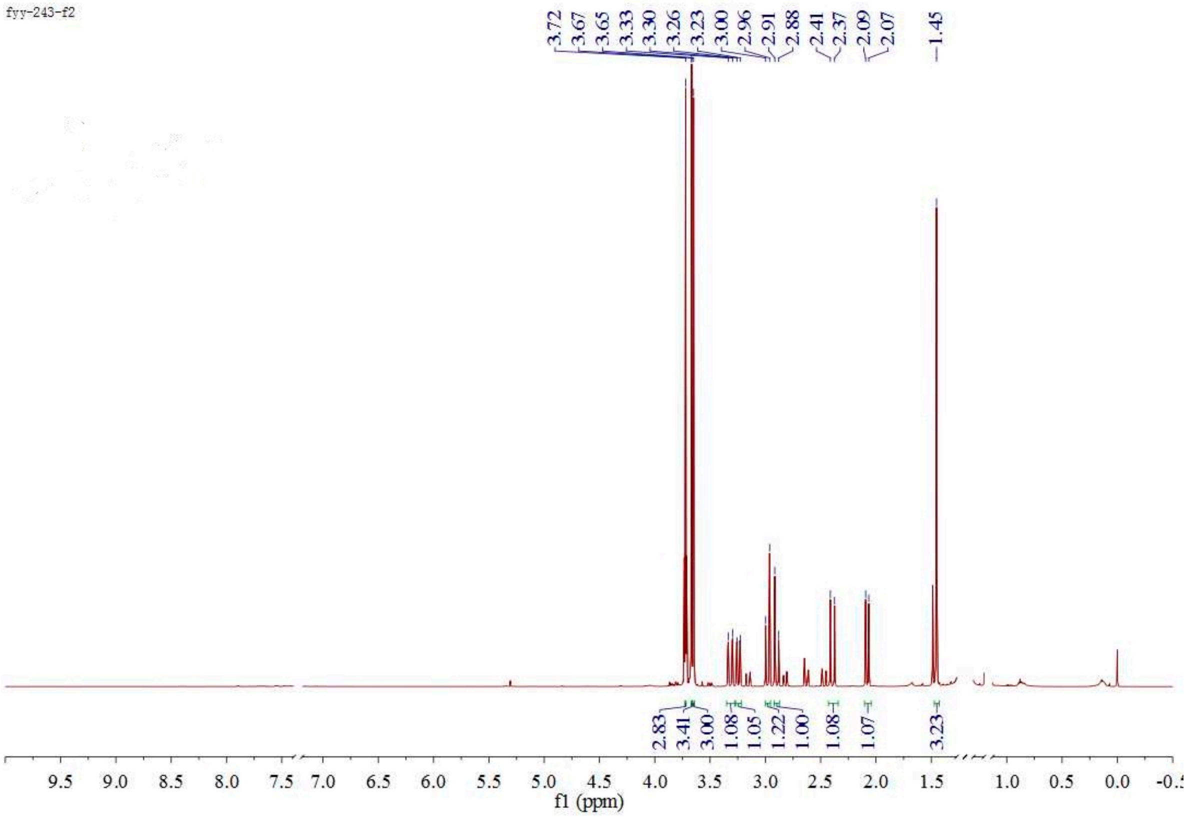

Figure S21. 1 ^{1}H NMR of *trans*-**3a**.

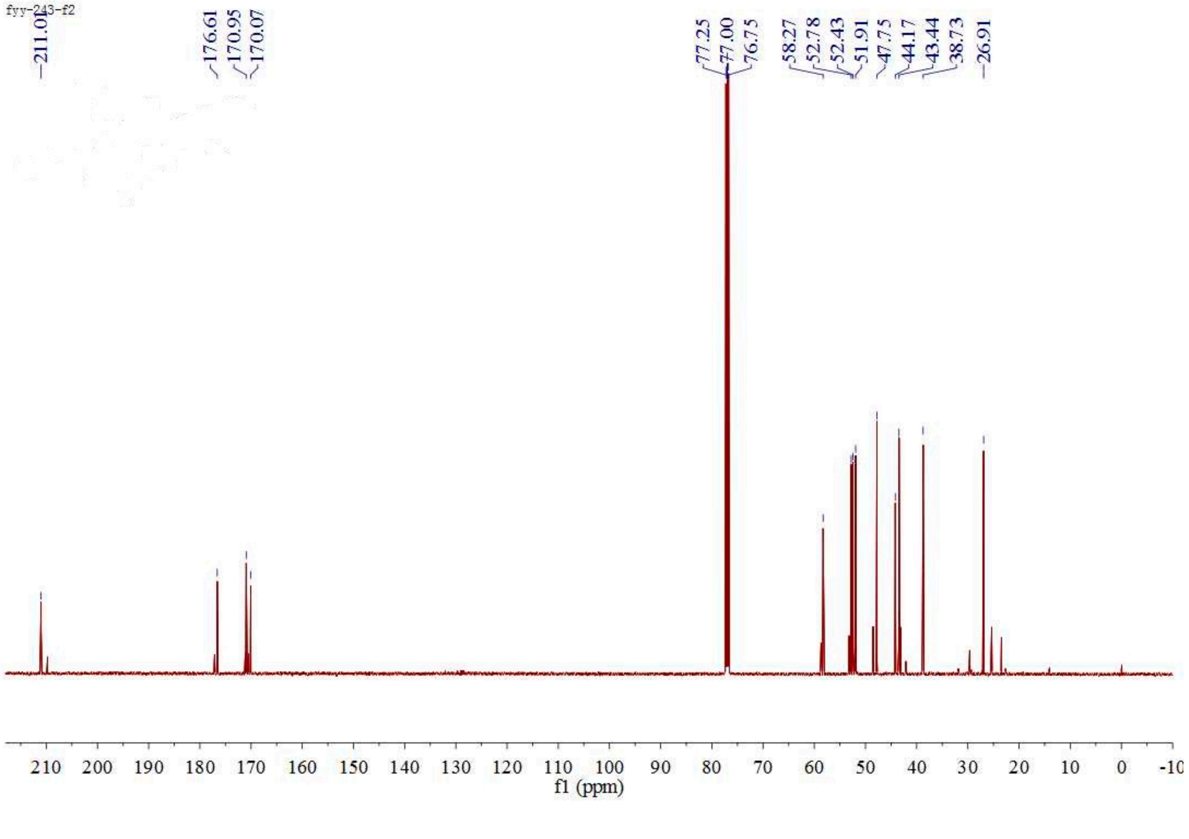

Figure S22. ^{13}C NMR of *trans*-**3a**.

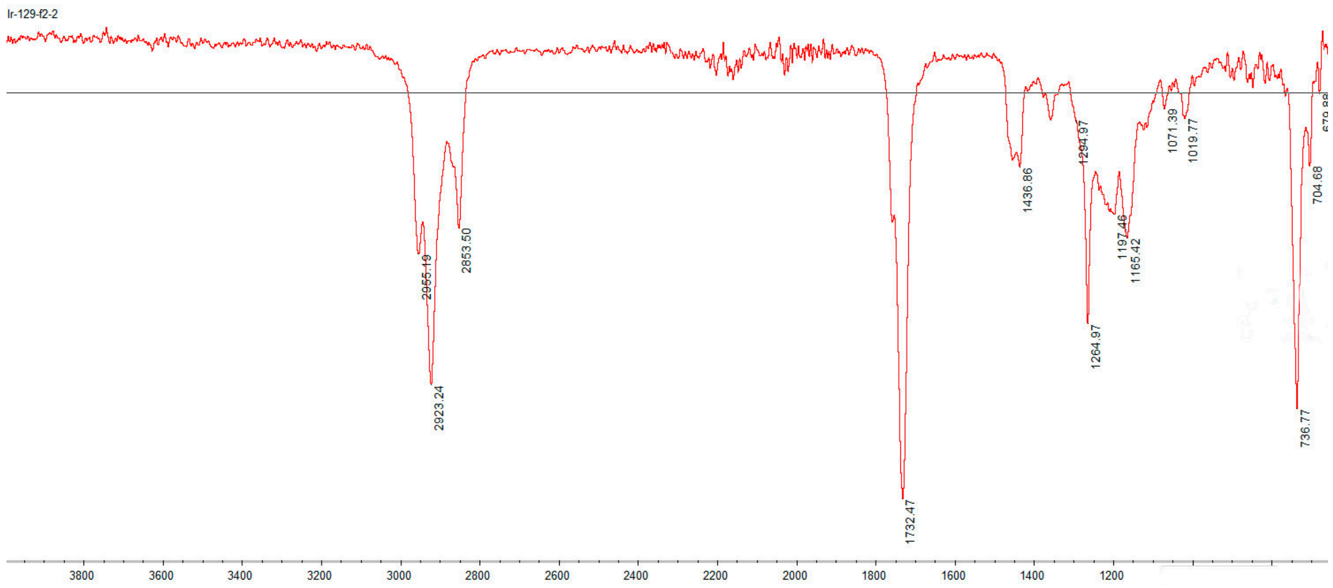

Figure S23. IR of *cis*-**2a**.

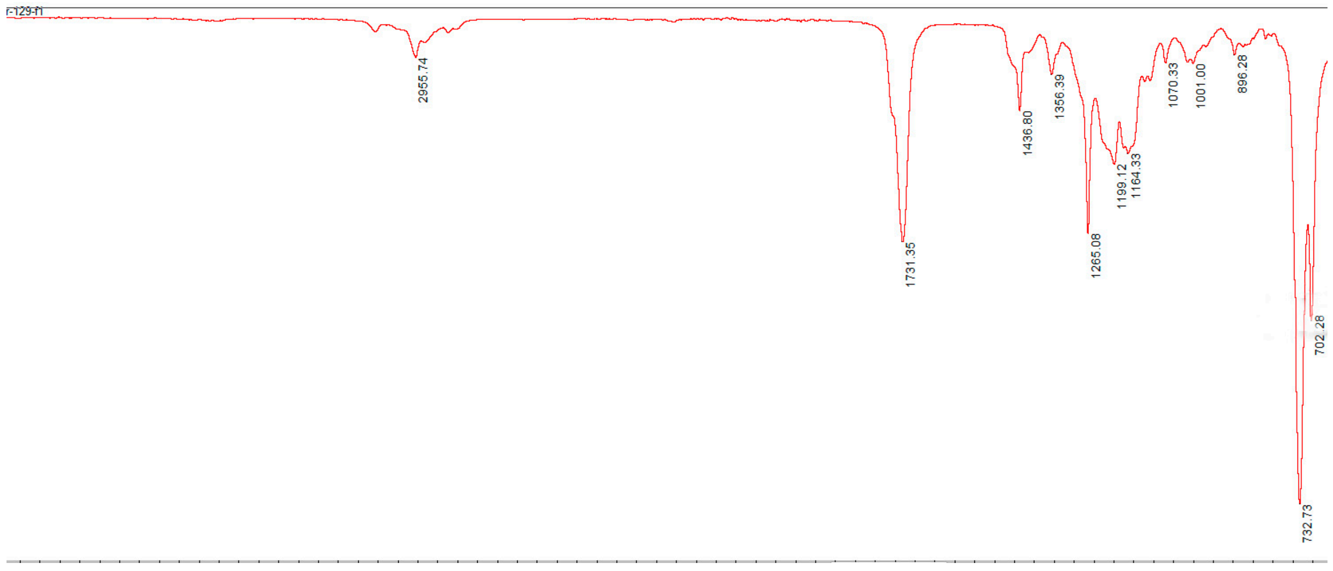

Figure S24. IR of *trans*-**2a**.

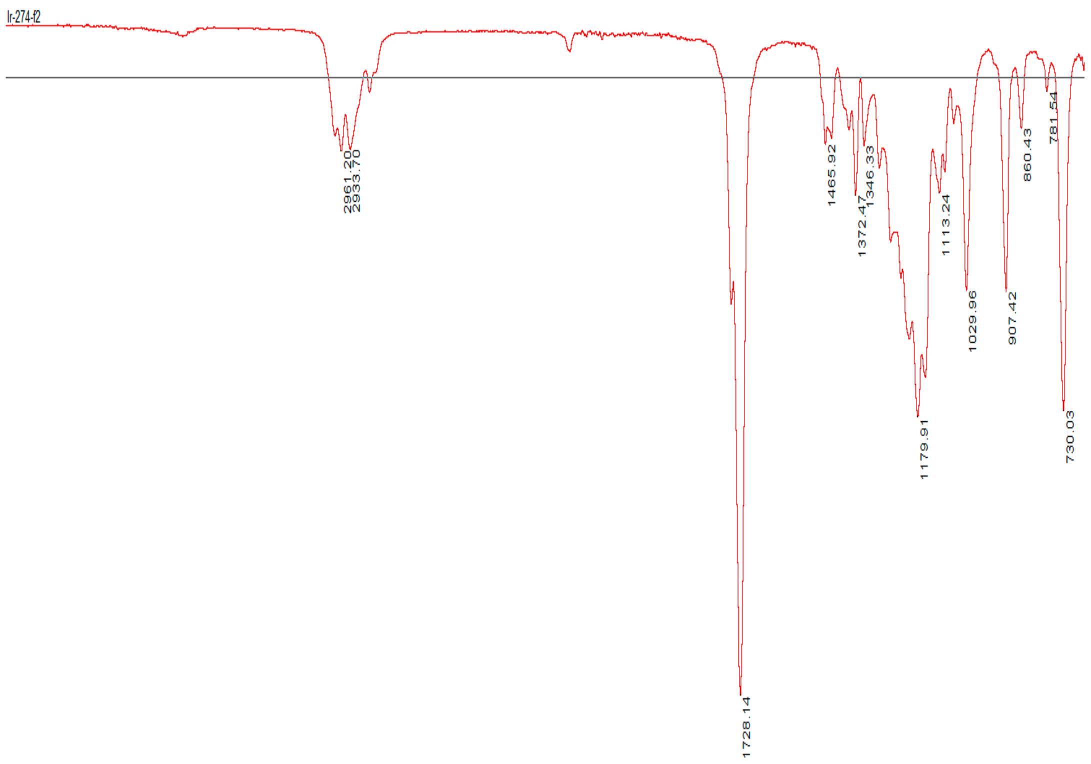

Figure S25. IR of *cis*-**2b**.

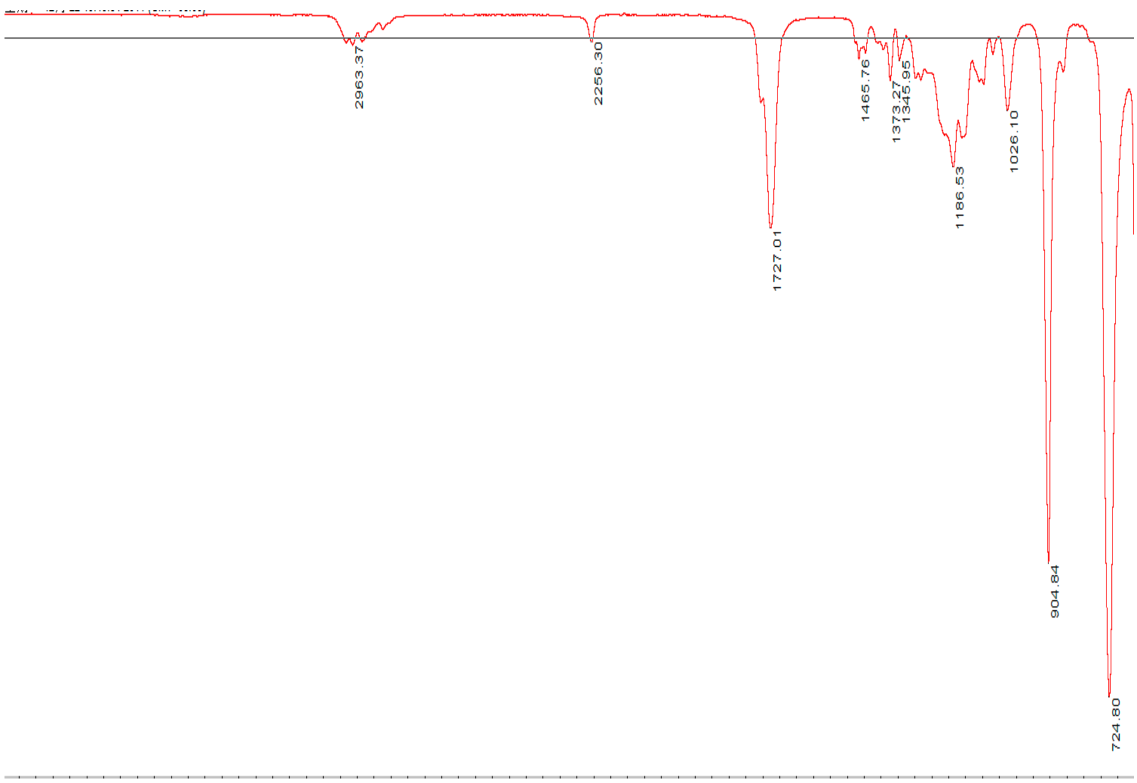

Figure S26. IR of *trans*-**2b**.

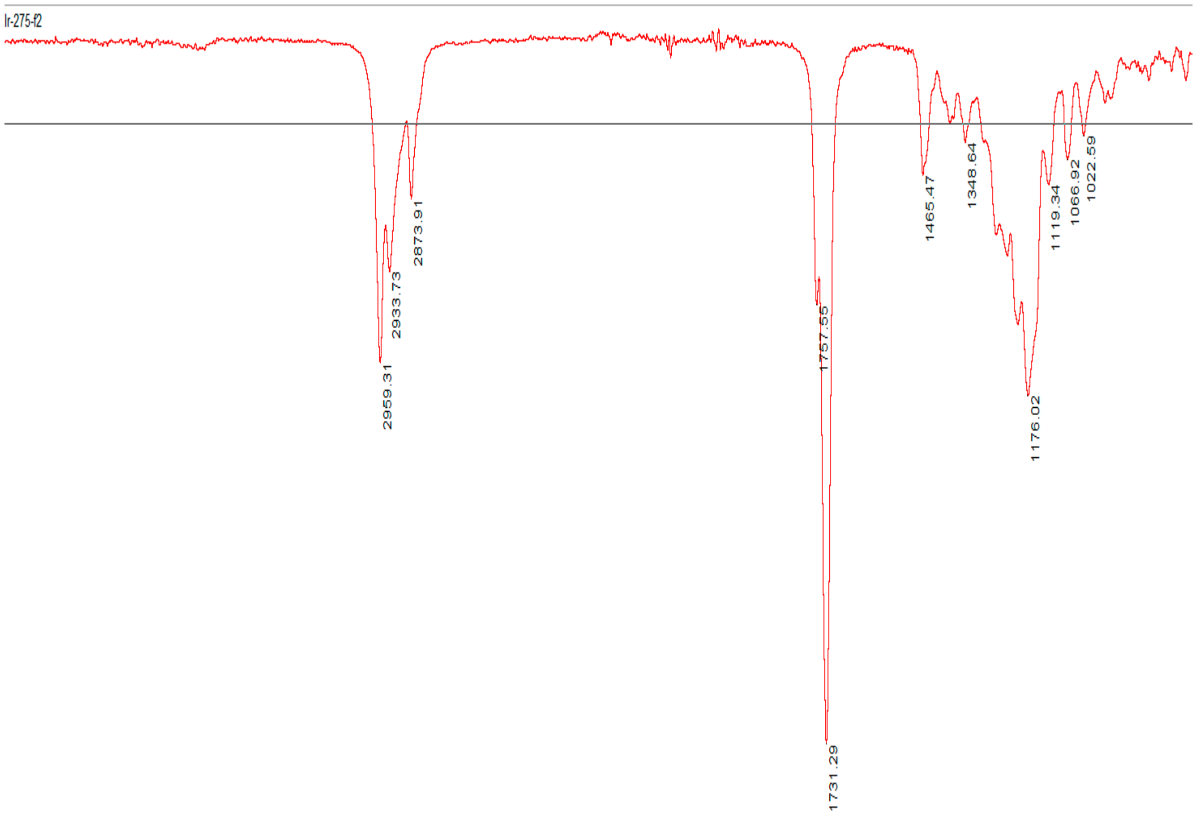

Figure S27. IR of *cis*-**2c**.

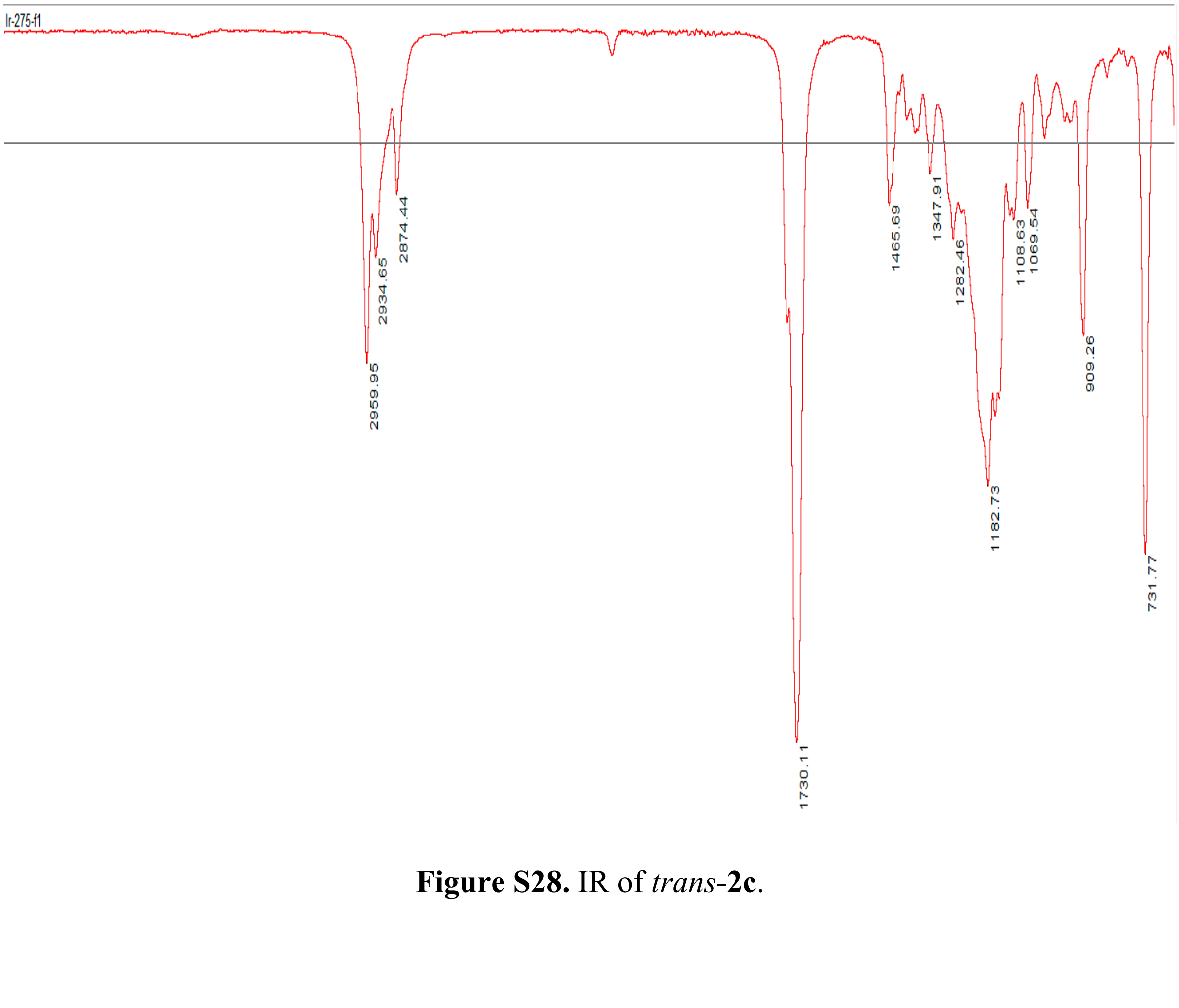

Figure S28. IR of *trans*-**2c**.

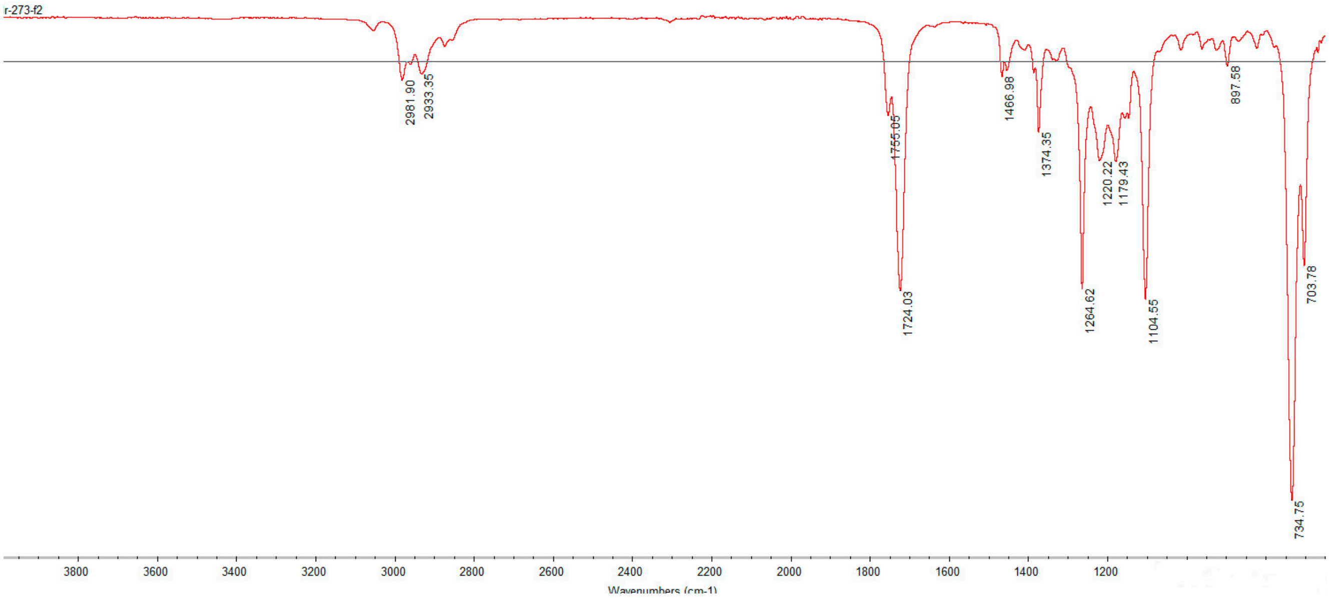

Figure S29. IR of *cis*-**2d**.

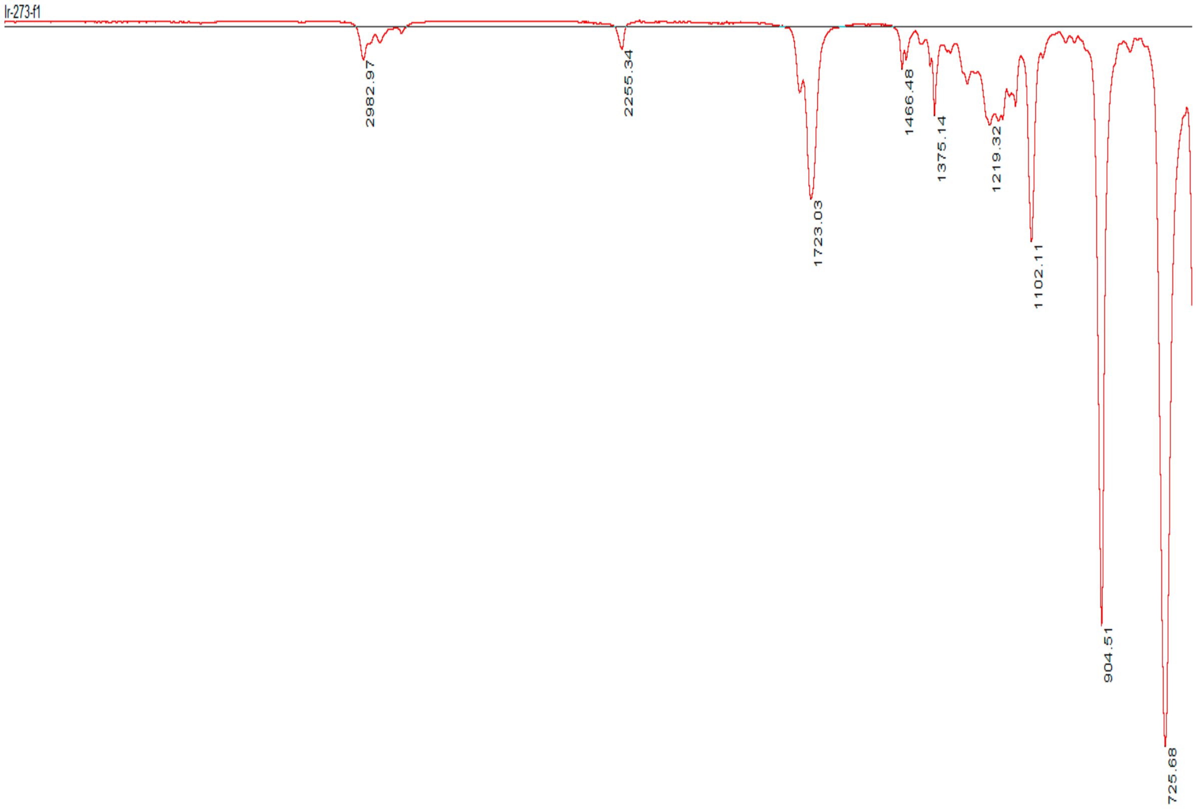

Figure S30. IR of *trans*-**2d**.
